# Keystone seabird may face thermoregulatory challenges in a warming Arctic

**DOI:** 10.1038/s41598-023-43650-5

**Published:** 2023-10-04

**Authors:** Melissa L. Grunst, Andrea S. Grunst, David Grémillet, Akiko Kato, Sophie Gentès, Jérôme Fort

**Affiliations:** 1 Littoral, Environnement et Sociétés (LIENSs), UMR 7266 CNRS-La Rochelle Université, 2 Rue Olympe de Gouges, 17000 La Rochelle, France; 2 CEFE, Univ Montpellier, CNRS, EPHE, IRD, Montpellier, France; 3 Percy FitzPatrick Institute of African Ornithology, University of Cape Town, Rondebosch, South Africa; 4 Centre d’Etudes Biologiques de Chizé, CEBC, UMR 7372 CNRS-La Rochelle Université, La Rochelle, France

**Keywords:** Climate-change ecology, Ecophysiology

## Abstract

Climate change affects the Arctic more than any other region, resulting in evolving weather, vanishing sea ice and altered biochemical cycling, which may increase biotic exposure to chemical pollution. We tested thermoregulatory impacts of these changes on the most abundant Arctic seabird, the little auk (*Alle alle*). This small diving species uses sea ice-habitats for foraging on zooplankton and resting. We equipped eight little auks with 3D accelerometers to monitor behavior, and ingested temperature recorders to measure body temperature (T_b_). We also recorded weather conditions, and collected blood to assess mercury (Hg) contamination. There were nonlinear relationships between time engaged in different behaviors and T_b_. T_b_ increased on sea ice, following declines while foraging in polar waters, but changed little when birds were resting on water. T_b_ also increased when birds were flying, and decreased at the colony after being elevated during flight. Weather conditions, but not Hg contamination, also affected T_b_. However, given our small sample size, further research regarding thermoregulatory effects of Hg is warranted. Results suggest that little auk T_b_ varies with behavior and weather conditions, and that loss of sea ice due to global warming may cause thermoregulatory and energic challenges during foraging trips at sea.

## Introduction

Global climate change is proceeding at unprecedented rates^1^, posing physiological and bioenergetic challenges for organisms^2–4^. Among direct effects of shifting weather regimes on animals, thermoregulatory challenges are particularly important^5–9^. Organisms are increasingly facing challenging thermal environments in the form of elevated temperatures, extreme heat events and stochasticity^1^. In addition, other environmental conditions, such as wind patterns and humidity affect heat exchange with the environment^10,11^, and may evolve under climate change^1^. Organisms are also facing shifts in resource distributions and changes in habitat structure, which may force changes in activity patterns and create thermoregulatory challenges that disrupt energy balance. For example, modified vegetation structure may not only affect the resource base, but also limit availability of shade, and scope for behavioral thermoregulation^12^. Furthermore, in the Arctic, which is warming ~ 4 times faster than other regions^1,13^, advanced melting and decreased extent of sea ice may mean longer commutes to favorable foraging areas^14^ and loss of an important resting substrate that provides thermal relief at sea^15^.

Compounding the challenges of climate change, organisms simultaneously face other environmental challenges, some of which are also linked to ecosystem engineering by humans. For example, anthropogenic activities have increased exposure to chemical contaminants, such as mercury^16^, and global warming has potential to exacerbate this threat^17–19^. Mercury (Hg) is a prevalent contaminant that reaches even remote polar regions via a repeated process of condensation and evaporation, persists in cold environments, and biomagnifies up marine food chains^16,20,21^. The methylated form, methylmercury (MeHg) is especially bioavailable and harmful to wildlife^22^. Exposure of animals to MeHg may be exacerbated by climate change^23^. For instance, conversion of inorganic Hg to MeHg is potentiated in warming oceans^24^. Hg contamination may interfere with body temperature (T_b_) regulation via endocrine disruption^25^, notably affecting production of thyroid hormones, which are central to thermoregulation^26^. In addition, Hg contamination could affect patterns of thermoregulation through effects on detoxification costs and resting metabolic rate^27–29^. Although few data specific to Hg are available, both hypo- and hyperthermic responses can occur in response to contamination^30^. Hypothermic responses are proposed to reflect an adaptive detoxification mechanism through which toxicity is reduced through facultative reduction in T_b_^30,31^. Modification in T_b_ due to contaminant exposure could limit thermoregulatory adjustments in responses to global climate change, making it more difficult for animals to conserve water and energy.

In this study, we used a suite of advanced techniques to evaluate potential for shifting environmental conditions and Hg contamination to affect activity-specific T_b_ regulation in a keystone Arctic seabird, the little auk (or dovekie, *Alle alle*). We fit eight free-ranging birds with internal T_b_ loggers, which were ingested by focal individuals, and recorded abdominal temperature as a proxy of core T_b_ through time. Birds were simultaneously equipped with miniaturized triaxial (3D) accelerometers that recorded body acceleration, allowing classification of activity budgets. We used data from an onsite weather station to gain insight into links between weather conditions and T_b_. Finally, we obtained blood samples to assess whether Hg contamination could affect T_b_ and limit adaptive plasticity, although given our small sample size, we lack the power to draw strong conclusions regarding effects of Hg contamination.

We generated a suite of predictions based on our knowledge of the behavior, morphology and energetics of the little auk. In general, we predicted that environmental conditions and activity would interact to affect mean levels and variation in core T_b_. More specifically, little auks have a high wing loading, resulting in extremely high energetic costs of flight^32^. Thus, we predicted that T_b_ would increase when birds were flying relative to during other activities, and that this increase would be magnified under conditions that reduce heat exchange between the body and environment or increase flight costs. In contrast, endothermic animals diving into cold polar waters face thermal challenge due to the high thermal conductance of water^33,34^. As a result, diving animals often allow T_b_ to fall below normothermic levels, which may facilitate aerobic dive capacity and limit energetic costs of heat loss to the environment^34,35^. Thus, we predicted that T_b_ would decline over the course of foraging episodes, and subsequently increase when birds were resting on sea ice. However, we recognized the potential that regional heterothermy, that is, variation in peripheral temperatures, especially in the appendages, might buffer changes in core T_b_ during diving, resulting in relative stability^35^. We also predicted that variation in T_b_ might increase in the context of thermal challenge, which in the Arctic is most commonly experienced in the context of cold stress, especially during resting periods, but which could also involve heat stress, especially during activity. Finally, we predicted that higher blood Hg concentrations might affect thermoregulatory capacity. More specifically, higher Hg concentrations could be linked to either higher mean T_b_, perhaps reflecting increased metabolic rates to support detoxification costs, or lower T_b_, perhaps reflecting suppression of thyroid hormones. In addition, elevated blood Hg could be associated with greater variation in T_b_, especially in the context of thermal stress. By adopting a multifaceted approach and evaluating specific predictions, we grant insight into how thermoregulatory dynamics may shift given concomitant exposure to multiple environmental stressors.

## Materials and methods

### Study system

Our study took place at a breeding population of little auks situated at Ukaleqarteq (Kap Höegh), East Greenland (70°44′N, 21°35′W). Little auks (~ 150 g) are the most abundant seabird in the high Arctic and breed in large colonies^36^. Both males and females incubate a single egg, and contribute equally to provisioning of the chick^37,38^. Little auks forage on copepods, and return to the colony from foraging sites at sea with prey items stored in a specialized gular pouch, which are then regurgitated for the chick^38^. Upon return to the colony, little auks can be captured and recaptured at or near the nesting sites in rock crevasses, facilitating fitting and retrieval of accelerometers and deployment of T_b_ loggers. Mean blood Hg levels in little auks at Ukaleqarteq in recent years fall into the low risk range for toxicological effects. However, past research documents negative effects on reproduction^39,40^ and body condition^14^, suggesting that despite relatively low Hg levels, there may be other effects on physiology and thermoregulation. Fieldwork adhered to the ASAB/ABS guidelines for use of animals in behavioral research, and was conducted in accordance with Greenlandic law. The Government of Greenland, Ministry of Environment and Nature, and Department of Fisheries, Hunting and Agriculture, approved research procedures and provided ethical clearance (permit: 2020-1006). The methodology and results of this study are reported in accordance with ARRIVE guidelines.

### Deployment of T_b_ loggers and accelerometers

During July 2020, we captured eight little auks outside nesting crevasses using a combination of lassos and noose carpets. We fit each bird with a T_b_ logger (BodyCap Anipill Core Body Temperature Ingestible Tablet; BMedical; ± 0.2 °C; 1.7 g; 17.7 × 8.9 mm; ~ 1% of mass), a data logging system for gastrointestinal temperature recording. These capsules are designed for use in birds, and have been used by a number of past studies on wild and free-ranging species^41,42^. Birds spontaneously ingested T_b_ loggers placed within the beak. Loggers recorded abdominal temperature every minute for periods of ca. 30 h. We remotely downloaded data from loggers via telemetry when the bird was within ~ 1 m. T_b_ loggers are assumed to be eliminated through defecation or regurgitation, and are not recovered. Focal individuals were simultaneously equipped with miniaturized triaxial accelerometers (Axy 4, Technosmart, 3 g, ~ 2% of mass), to record body acceleration. We attached accelerometers to the central breast at the level of the sternum using Tesa® tape. Accelerometers recorded data at a frequency of 50 Hz (50 readings per second). We marked birds with color rings to facilitate identification and recapture within ~ 4 days, upon which we retrieved the accelerometer. Deployment dates fell within nine days during the mid-late chick rearing phase [July 22–30]. All birds were breeding adults, but sex was not determined for this study, as the remaining volume of blood was conserved for other physiological assays. The thermoregulatory physiology of male and female little auks during the nestling stage is likely to be similar, as the sexes are monomorphic in coloration, overlap extensively in size (males may be slightly larger), and share equally in breeding duties^37,38^. Nevertheless, we recognize lack of knowledge of sex as a limitation to our work. A portable HOBO H21-USB weather station at the study site recorded weather conditions (every 1-min), including ambient temperature (T_a_; °C), relative humidity (RH; %) and wind speed (V; m/s). Conditions measured at the weather station were taken as a proxy of environmental conditions at both the breeding site and at-sea foraging grounds (as a caveat, little auks can forage up to ~ 100 km from the colony^43^).

### Analysis of accelerometry and T_b_ data

We used Igor Pro 8.04 (64-bit; WaveMetrics) to classify data on triaxial acceleration into behavioral states (see details in^15^). In brief, we used k-clustering analysis applied to acceleration axes, followed by application of a custom-written script, which utilized output from the clustering analysis and surface temperature data. Behavioral states identified were: flying, diving, on the water surface, on sea ice, and at the colony. We proceeded to determine whether time spent on the water was part of a foraging bout (i.e. inter-dive interval), or represented time resting on the water. To this end, we determined the dive bout ending criterion, using R package diveMove^44^, which applies the methods of^45^ and^46^ for identification of behavioral bouts. Using the standard method of classification, based on absolute duration of behavioral bouts (i.e. inter-dive intervals), the bout ending criteria derived was 307.1 s. Consequentially, we ended diving bouts if time spent on the water exceeded this value, and classified these intervals as time resting on the water. Time resting on the water additionally encompassed intervals of time on the water that were not between dives. We combined time engaged in diving and inter-dive intervals into a single behavioral category, representing active foraging. Thus, final behavioral categories were: actively foraging (hereafter also “diving”), flying, at the colony, on sea ice, and resting on the water. For each T_b_ measurement, we determined the corresponding behavioral state by aligning time stamps from the T_b_ and behavioral (accelerometer) data in Microsoft Excel 16.16.27.

### Mercury contamination: sampling and analysis

We obtained ~ 0.2–0.5 ml blood samples from the brachial vein after recapture of birds to retrieve accelerometers, which minimized stress during the experimental period. Blood samples were centrifuged for 10 min at 3500 rpm to separate plasma from red blood cells (RBCs), which were stored in 70% ethanol. After evaporation of ethanol, RBCs were freeze dried for 48 h and homogenized prior to analysis for total Hg (hereafter Hg) concentrations. Total Hg serves as a proxy for highly toxic MeHg, since most of the Hg in bird blood, feathers and eggs is MeHg^47^. Samples were analyzed in duplicate using an Advanced Mercury Analyser spectrophotometer (Altec AMA 254) at the Institute Littoral Environnement et Sociétés (LIENSs)^48^. The standard deviation between duplicates was < 10%. We used TORT-3 as a standardized reference material (CRM; Lobster Hepatopancreas Tort-3; NRC, Canada; [Hg] = 0.292 ± 0.022 µg g^−1^ dry weight (dw)) and performed a blank before initiating measurements on samples. The limit of detection for Hg and mean ± SD of Tort-3 measurements were 0.005 µg g^−1^ dw and 0.302 ± 0.004 µg g^−1^ dw (*N* = 8; replicates), respectively.

### Statistical analysis

We conducted statistical analyses in R 3.6.1^49^. We used generalized additive mixed effect models (GAMMs) in R package mgcv^50,51^ to assess whether T_b_ varied with behavior, environmental conditions, or time of day. For this model, we used each observation of T_b_. We used the corAR1 correlation structure in package nlme to account for temporal autocorrelation^52^, included individual ID and behavioral bout (nested within individual ID) as random effects, and incorporated two non-linear smooth terms. The first non-linear smooth term tested for non-linear variation between T_b_ and time in each behavioral state. To this end, we used a cubic regression spline (specified as bs = ”cr”) with the degree of smoothness set to k = 50 (which minimized AICc and increase the R^2^ relative to models with lower k). We used the “by” call within the smoothing function to test for unique non-linear relationships within each behavioral state. For the second non-linear smooth term, we used a cyclic cubic regression spline (specified as bs = ”cc”) to test for variation in T_b_ with time of day. We also included two-way interactions between behavioral state and: (1) T_a_, (2) RH, (3) wind speed, and (5) Hg concentrations. We removed interactions with *P* values > 0.059 from models, followed by elimination of main effects above *P* > 0.05. We standardized continuous predictor variables to a mean of zero and standard deviation of one to facilitate interpretation of main effects when including interactions in models^53^. We used package emmeans^54^ for pairwise comparisons between interaction terms (function emtrends) and differences in T_b_ between behavioral states (function emmeans).

To more thoroughly explore differences in how T_b_ changes with time when birds are engaged in different behaviors, we calculated change in T_b_ (deltas) for each behavioral bout as: ΔT_b_ = T_b,end_-T_b,start_; where T_b,end_ = T_b_ at the last time point recorded in that behavioral state and T_b,start_ = T_b_ at the first time point recorded. We used a linear mixed effects model in nlme to compare ΔT_b_ across behavioral states, while including the length of the time interval in the model. We extracted and plotted predicted values from models using function ggpredict within the ggeffects package^55^. In addition, to evaluate modulators of the effect of time of day on T_b_, we constructed GAMMs predicting T_a_ and RH from time of day, using the same random structure as above.

We also assessed whether between minute variation in T_b_ differed between behavioral states by calculating the absolute value of the difference between subsequent measurements of T_b_, and constructing models with the same structure described for mean T_b_. Values could not be calculated for time points at the beginning of behavioral intervals, so these rows were dropped from the analysis.

## Results

### Predictors of little auk T_b_ across behavioral states

Mean ± SD T_b_ of little auks was 41.0 ± 0.55 °C and showed significant, but low magnitude differences between behaviors (Table 1; see Table S1 for full GAMM, including non-significant effects). Estimated marginal means (EMM ± SE [95% CI]) for T_b_ were significantly lower for diving (40.7 ± 0.092 [40.5, 40.8] °C) relative to in other behaviors (*P* < 0.001 in all cases; Table S1for pairwise comparisons), but did not differ between flying (41.55 ± 0.092 [41.4, 41.7] °C), at the colony (41.4 ± 0.089 [41.2, 41.6] °C), on sea ice (41.4 ± 0.088 [41.2, 41.6]°C), or on the water (41.4 ± 0.105 [41.2, 41.6]°C) (Table S2 for pairwise comparisons).

**Table 1:** Minimum adequate GAMM for body temperature (T_b_ ; °C) in little auks as a function of behavioral state, weather conditions, and time.

Variables	*β* ± SE	*t*	*P* > *(|t|)*	*F*	*P(*> *F)*
**Parametric coefficients**					
Intercept	40.6 ± 0.464	87.414	< 0.001		
Flying^a^	0.922 ± 0.496	1.857	0.063	7.271	< 0.001
Colony	0.817 ± 0.462	1.767	0.077		
Ice	0.325 ± 0.469	0.692	0.489		
Water	0.686 ± 0.495	1.385	0.166		
Wind speed (m/s)	− 0.016 ± 0.007	− 2.407	0.016	5.792	0.016
Relative humidity (%)	0.063 ± 0.013	5.028	< 0.001	25.3	< 0.001
Flying × wind	0.028 ± 0.010	2.836	0.004	2.345	0.052
Colony × wind	0.020 ± 0.010	2.012	0.044		
Ice × wind	0.021 ± 0.010	2.167	0.030		
Water × wind	0.016 ± 0.011	1.405	0.159		

	*edf*	*Ref.df*	*F*	*P(*> *F)*	
**Approximate significance, Smoothed terms**					
s(Time behavior): Diving	9.434	9.434	67.2	< 0.001	
s(Time behavior): Flying	5.991	5.991	10.1	< 0.001	
s(Time in behavior): Colony	5.933	5.933	22.5	< 0.001	
s(Time in behavior): Ice	5.097	5.097	21.2	< 0.001	
s(Time in behavior): Water	2.110	2.110	0.738	0.446	
s(Time of day)	6.467	18.0	3.940	< 0.001	
R-adjusted	0.212	N	16,405, 8		

^a^Relative to diving.

**Figure 1:**
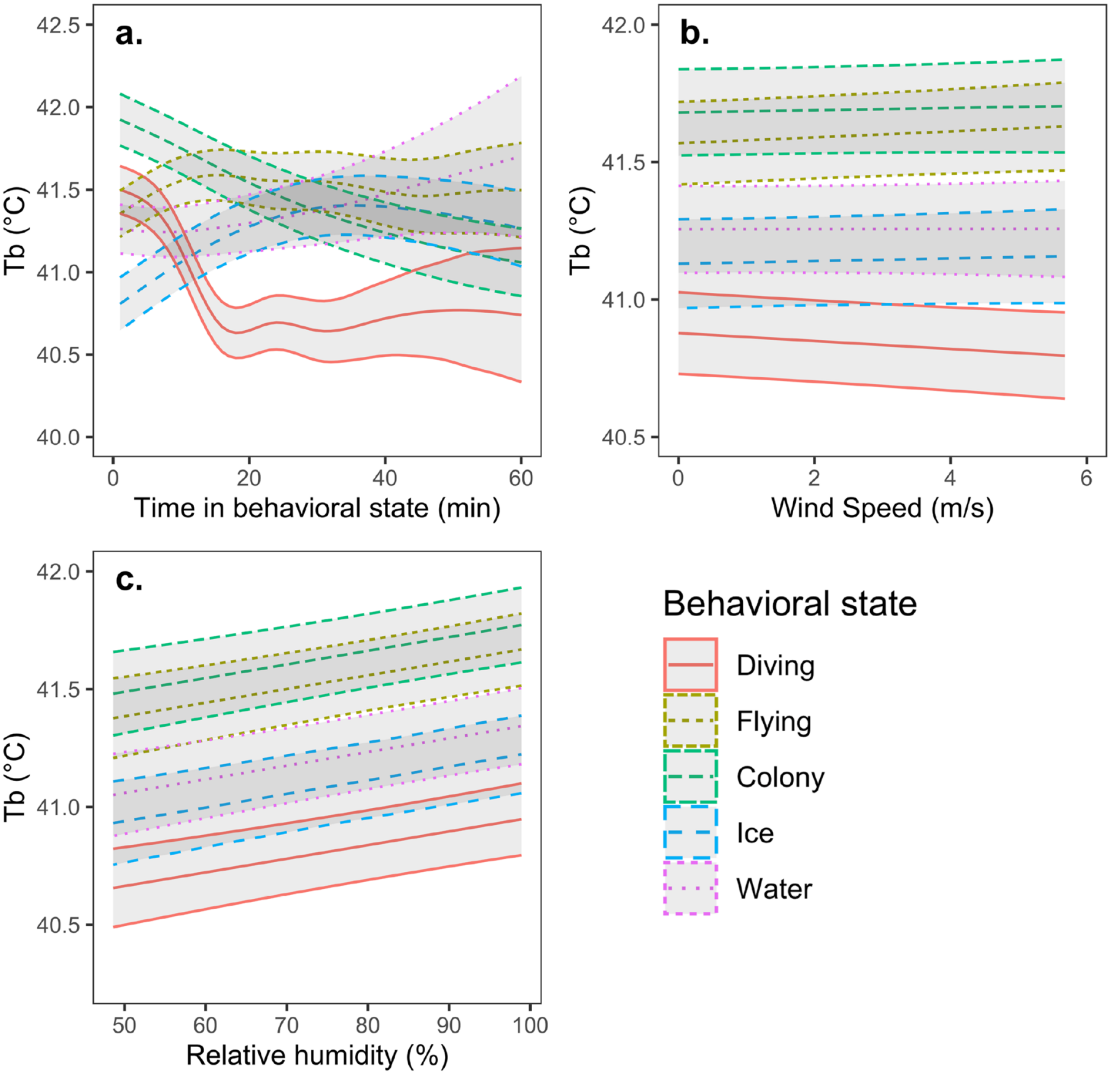
Relationships between T_b_ (°C) of little auks predicted from the GAMM and (**a**) time within the behavioral state (min), (**b**) wind speed (m/s), and (**c**) Relative humidity (%). Shaded regions show 95% CIs.

**Figure 2:**
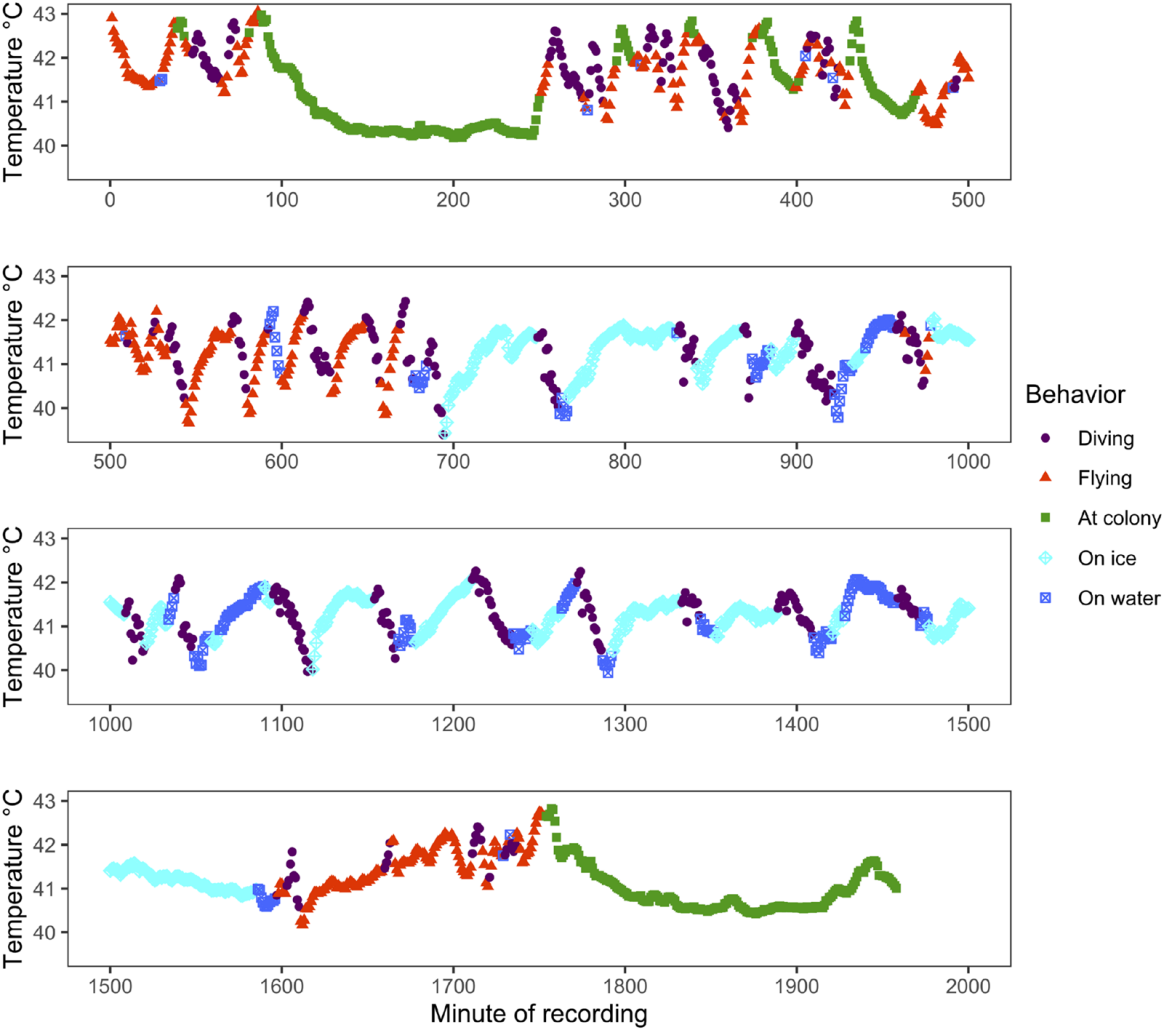
Body temperature (T_b_) through time color coded with respect to behavioral state for one little auk (LIAK20EG19) from the Ukaleqarteq, East Greenland, population. Note rebounds in T_b_ when on sea ice following declines while diving in cold Arctic waters. T_b_ also generally increases with time flying and declines with time at the colony.

The minimum adequate model predicting T_b_ included non-linear relationships with time spent in each of the behaviors, with the exception of time spent on the water surface, for which the relationship was non-significant (Table 1). T_b_ decreased with the amount of time spent diving up to ~ 19 min, from ~ 41.5 to 40.6 °C, after which predicted T_b_ plateaued (Fig. 1a). There was also a more gradual decrease in T_b_ after birds arrived at the colony up to ~ 90 min, from 41.9 to 40.9 °C (Fig. 1a). On the other hand, T_b_ increased the longer birds spent flying to ~ 15 min, from ~ 41.3 to 41.6 °C°, before leveling off or declining slightly (Fig. 1a). T_b_ also displayed an increase for the initial 30 min when birds were on the sea ice, from ~ 40.8 to 41.4 °C, after which T_b_ leveled off or declined slightly (Fig. 1a). Figure 2 shows a trace of T_b_ through time for one focal individual. See Fig. S1–S7 for other birds.

The overall ΔT_b_ for diving was negative, with the 95% CI not overlapping zero (EMM ± SE [95% CI] = − 0.312 ± 0.026 [− 0.374, − 0.251] °C), and was lower than all other ΔT_b_ (Table S3 for pairwise comparisons). The ΔT_b_ for flying and on sea ice were positive, with Cis not overlapping zero (EMM ± SE [95% CI] = 0.146 ± 0.029 [0.077, 0.215] °C; 0.335 ± 0.045 [0.228, 0.443] °C), and were higher than all other ΔT_b_, with ΔT_b_ for sea ice also greater than that of flying (Table S3 for pairwise comparisons). The overall ΔT_b_ for at the colony and on the water were negative, and positive, respectively, but did not significantly differ from each other or zero (Estimate marginal mean ± SE = − 0.074 ± 0.044 [− 0.179, 0.031] °C; 0.0002 ± 0.038 [− 0.089, 0.089] °C, respectively).

With respect to environmental effects, there was a significant interaction between behavioral state and wind speed in predicting T_b_ (Table 1). T_b_ tended to increase with wind speed when birds were flying, but not in other behavioral states (Table 1; Fig. 1b; Table S4 for pairwise comparisons). In addition, T_b_ was positively related to RH (Table 1; Fig. 1c), independent of behavioral state (Table S1). T_a_ was unrelated to T_b_, either independently, or in interaction with behavioral state (Table S1).

**Figure 3:**
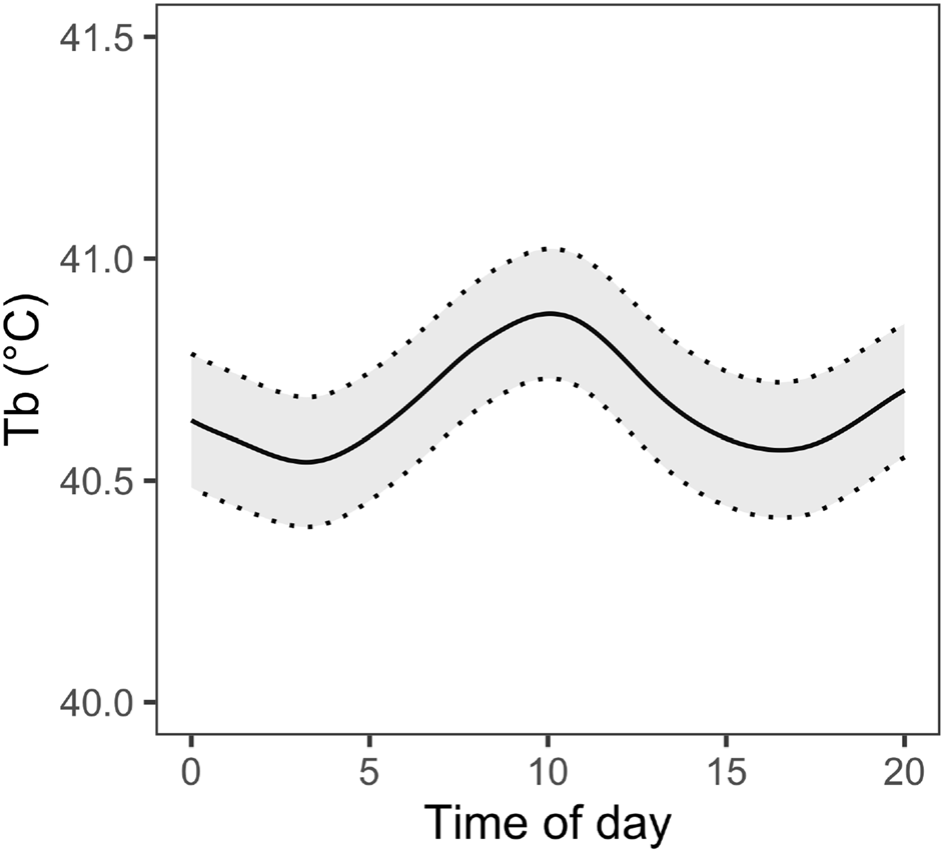
Relationship between T_b_ (°C) of little auks predicted from the GAMM and time of day. Shaded regions show 95% Cis.

Finally, the best model predicting T_b_ included a non-linear effect of time of day on T_b_ (Table 1; Fig. 3), with bimodal peaks in the late morning (~ 10:00) and around 20:00, and the lowest values in the early morning (~ 4:00 am) and around 16:00 (Fig. 3). When examining potential environmental effects underlying this relationship, we found that there was a non-linear, but unimodal, relationship between time of day and T_a_, with a peak around ~ 16:00 (edf = 7.33, *F*_8_ = 175, *P* < 0.001; Fig. S8). Similarly, RH was non-linearly related to time, and showed a reverse pattern to T_a_, with a nadir around ~ 16:00 (edf = 7.30, *F*_8_ = 203, *P* < 0.001; Fig. S9).

### Predictors of between minute variation in T_b_

**Figure 4:**
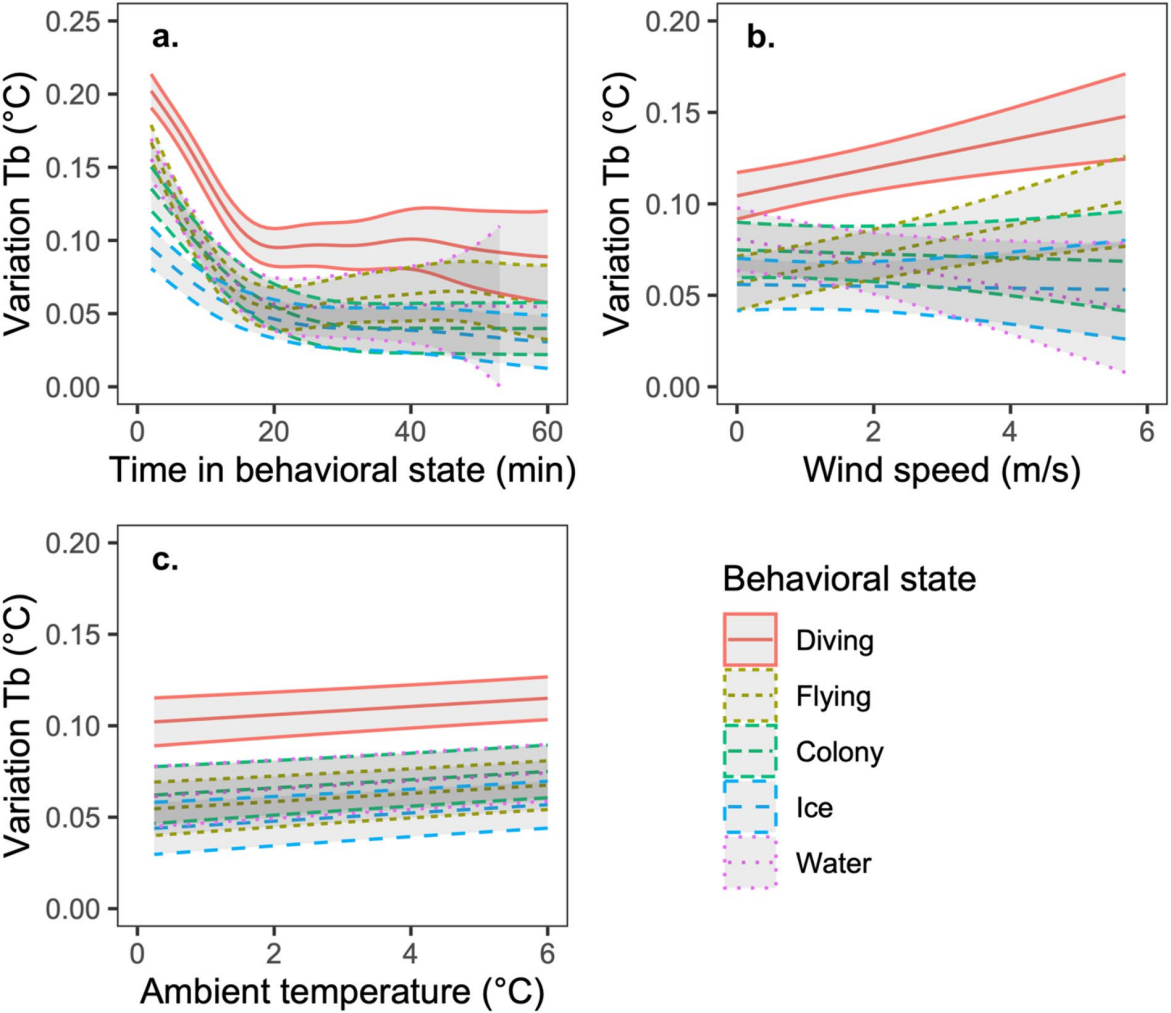
Between minute variation in little auk body temperature (|T_b1_-T_b1+1_|) (°C) in different behavioral states as a function of (**a**) time in behavioral state, b) wind speed, and (**c**) ambient temperature (°C). Plots show predicted values from GAMMs, with shaded regions representing 95% CIs.

Mean ± SD between minute variation in T_b_ (|T_b1_-T_b1+1_|) of little auks was 0.09 ± 0.11 °C (range: 0–1.5 °C), and showed low magnitude, significant variation between behavioral states (Table 2; Table S5 for full model). The EMM (± SE) was highest when birds were foraging at sea (0.097 ± 0.008 [0.080, 0.113] °C), followed by flying (0.060 ± 0.008 [0.044, 0.077] °C), on the water (0.055 ± 0.011 [0.033, 0.078] °C), at the colony (0.041 ± 0.008 [0.024, 0.057] °C) and on sea ice (0.040 ± 0.007 [0.025, 0.054] °C), but the only significant differences were between diving and all other behavioral states (Table S6 for pairwise contrasts). There were significant non-linear relationships between time in the behavioral state and between minute variation in T_b_ (Table 2); which involved early decreases with time in the behavioral state for all behaviors, before leveling off (Fig. 4a). This decrease was steepest when birds were resting on the water, diving, or in flight (all with a slope of ~ − 0.007 °C min^−1^ in the first ~ 15 min), and slightly more gradual at the colony (slope of ~ − 0.004 °C min^−1^ in the first ~ 25 min) and on the sea ice (slope of ~ − 0.002 in the first ~ 15 min) (Fig. 4a).

**Table 2:** Minimum adequate GAMM predicting between minute variation in body temperature (|T_b1_-T_b1+1_|) (°C) in little auks as a function of behavioral state and weather conditions.

Variable	*β* ± SE	*t*-value	*P* ( >|t|)	*F*	*P* (> F)
**Parametric coefficients**					
Intercept	0.155 ± 0.036	4.240	< 0.001		
Flying	− 0.039 ± 0.053	− 0.727	0.467	1.701	0.147
Colony	− 0.076 ± 0.037	− 2.069	0.039		
Ice	− 0.090 ± 0.038	− 2.366	0.018		
Water	− 0.067 ± 0.081	− 0.821	0.412		
Wind speed (m/s)	0.008 ± 0.002	3.372	0.001	11.373	< 0.001
Temperature (°C)	0.006 ± 0.002	3.882	< 0.001	15.074	< 0.001
Flying × wind	0.0002 ± 0.004	0.077	0.939	5.023	< 0.001
Colony × wind	− 0.010 ± 0.004	− 2.598	0.009		
Ice × wind	− 0.010 ± 0.004	− 2.317	0.021		
Water × wind	− 0.016 ± 0.005	− 3.415	< 0.001		

	*edf*	*Ref.df*	*F*	*P* (> F)	
**Approximate significance, Smoothed terms**					
s(Time behavior):Diving	6.315	6.315	66.68	< 0.001	
s(Time behavior):Flying	6.688	6.688	50.26	< 0.001	
s(Time behavior):Colony	11.58	11.58	15.75	< 0.001	
s(Time behavior):Ice	5.150	5.150	16.97	< 0.001	
s(Time behavior):Water	3.348	3.348	40.46	< 0.001	
R^2^-adjusted	0.234	**N**	15,136, 8

With respect to environmental effects, there was an interaction between wind speed and behavioral state in predicting between minute variation in T_b_ (Table 2; Fig. 4b). Variation in T_b_ increased with wind speed when birds were diving and flying but did not vary significantly with wind speed in the other behavioral states (Table S7 for pairwise comparisons). There was a positive correlation between T_a_ and variation in T_b_ (Table 2; Fig. 4c) independent of behavioral state (Table S5). When T_a_ was also in the model, RH was unrelated to variation in T_b_ (Table S5). However, when T_a_ was removed, RH was negatively related to variation in T_b_ (*β* ± SE = − 0.006 ± 0.002, *T* = − 3.42, *P* = 0.006), independent of behavioral state (Table S5).

Between minute variation in T_b_ did not vary with time of day (Table S5).

### Mercury contamination

There was no significant relationship between Hg contamination and T_b_ (Table S1) or between minute variation in T_b_ (Table S5). The mean ± SE of Hg concentrations in RBCs was 1.290 ± 0.031 µg g^−1^ dw [range: 1.030–1.746 µg g^−1^ dw], which assuming 79% blood moisture content is equivalent to 0.271 ± 0.007 µg g^−1^ ww [range: 0.216–0.367 µg g^−1^ ww], and falls within the low risk range for toxicological effects (0.2–1.0 µg g^−1^ ww^47^; calculated for whole blood, but comparable to levels in RBCs). Table 3 lists the Hg concentrations in RBC of each focal bird in dw and ww equivalents.

**Table 3:** Hg concentrations µg g^−1^ in dw and ww measured in the RBCs of focal birds breeding at Ukaleqarteq, East Greenland.

Focal individual	[Hg] µg g^−1^ dw, ww
LIAK20EG09	1.030, 0.216
LIAK20EG12	1.350, 0.284
LIAK20EG14	1.038, 0.218
LIAK20EG17	1.334, 0.280
LIAK20EG19	1.216, 0.255
LIAK20EG24	1.075, 0.226
LIAK20EG25	1.746, 0.366
LIAK20EG28	1.527, 0.321

## Discussion

By employing advanced biologging approaches, we demonstrate that the T_b_ of a free-ranging seabird is sensitive to variation in activity patterns and environmental conditions, and suggest thermoregulatory challenges that may arise under climate change scenarios. In particular, results suggest that the evolving nature of the Arctic cryosphere, especially sea ice extent and coverage, may not only alter foraging conditions for little auks^14,56,57^, but also affect thermoregulatory dynamics during foraging trips at sea. There was no evidence that Hg contamination modifies the T_b_ of little auks, but our results regarding potential toxicological effects on thermoregulatory capacity are limited by a small sample size. Thus, further research is called for in this area.

Changes in T_b_ with activity were generally as expected. T_b_ declined while little auks were foraging in cold waters. This decline may facilitate aerobic capacity and limit heat loss, but also reflects thermoregulatory challenge^34,58,59^. In addition, declines in T_b_ while foraging, and high variability in T_b_, may arise from ingestion of cold prey. Indeed, ingestion-linked declines in T_b_ have been used to identify feeding events^60^, but our data lacked resolution to achieve this end. In another Alcid, Brünnich's guillemots (*Uria lomvia*), T_b_ declined over sequential diving bouts^35^. However, this decline occurred during periods of resting on the water. During dives themselves, T_b_ increased and the peripheral temperature declined^35^. This pattern contrasts to T_b_ declines during diving observed in some penguin species^59,61,62^, and may reflect peripheral vasoconstriction and high wing beat frequency that generates heat^35^. Our data could not separate changes in T_b_ during dives and inter-dive intervals. Thus, a similar dynamic could be occurring in little auks.

Also as expected, T_b_ was highest when little auks were flying, although the estimated marginal mean was not significantly different than when birds were on sea ice, at the colony, or on the water. Furthermore, T_b_ increased during flight before leveling off, reflecting heat generated by intense physical activity. Indeed, little auks have high wing loadings and flight costs (~ 7.24 × BMR^32^), which is expected to generate substantial amounts of heat and elevate T_b_.

T_b_ was also high when little auks arrived at the colony. T_b_ then declined before leveling off. This decrease in T_b_ may reflect decreases following commuting flights between foraging sites and the colony, with a decline in diet-induced thermogenesis (i.e. heat production associated with digestion, also referred to as specific dynamic action^63^), also potentially contributing. T_b_ of little auks at the colony was also relatively invariable, which could suggest that the colony serves as a thermal refuge for little auks. During the current study, we did not detect evidence that little auks were subject to thermal stress on land. The upper critical temperature (UCT) of little auks is ~ 20°C^64^, a temperature not reached during the relatively cool 2020 breeding season. However, an air temperature of 20 °C was exceeded twice during July 2021, and captured birds were observed to rapidly exhibit sign of overheating (*unpublished data*). The operative temperature of little auks may be further elevated by solar radiation off the rocks, especially given their black plumage coloration^9^. Thus, with ongoing climate change, heat stress at the colony may eventually pose a threat, especially in the context of stress from predation pressure, which can activate flight-fight responses, or social interactions, which have been shown to elevate T_b_ in a range of animal species^65–67^. Indeed, due to their Arctic habitat and amphibious live style, little auks have evolved a low thermal conductance, which conserves heat and energy under cold conditions, but creates a challenge for heat dissipation^64^.

As predicted, little auks’ T_b_ increased substantially on sea ice following declines during diving episodes, before leveling off, suggesting that loss of sea ice as a resting substrate may elevate thermoregulatory costs, negatively affecting energy balance. T_b_ was initially low when little auks first emerged on the sea ice, which could reflect that birds exit the water when T_b_ falls below a threshold, triggering birds to cease foraging activity^35^. For the first 30 min, T_b_ on sea ice increased by an average of ca. 0.02 °C min^−1^, while during foraging T_b_ dropped by an average of ca. −0.05 °C min^−1^ for the first 19 min. Thus, the rate of T_b_ loss while foraging was greater than the rate of gain on ice, but given that the average ice bout lasted ca. 25 min and the average foraging bout ca. 9 min, time spent on ice would allow for recovery of T_b_. On the other hand, there was no significant relationship between time resting on the water and T_b_, and although positive, the ΔT_b_ of birds resting on water surface was not significantly different than zero, suggesting little potential for recovery of T_b_ after foraging bouts. Furthermore, the lowest variation in T_b_ occurred when birds were on sea ice, and the highest during diving behavior, suggesting that resting on sea ice plays an important role in allowing birds to restore and maintain normothermic temperatures after thermally challenging foraging bouts.

In the context of climate change, sea ice loss may have energetic and thermoregulatory implications, as birds are forced to instead rest on the water, which has ~ 25 × higher thermal conductivity than air^33,68^. In auks, compression of air space in feathers while diving reduces insulative properties, further facilitating heat exchange with the environment^69^. The costs of resting both in the air and on the water may be reduced by warming temperatures^70^. However, the thermal neutral zone, outside of which metabolic rate must be elevated to maintain T_b_, has been shown to be considerably narrower for seabirds resting on the water relative to when resting in air, with metabolic rate also increasing more steeply below the lower critical temperature (LCT) in some species^71^. For example, in Brünnich’s guillemot, the LCT in air and water are 2 and 16 °C, respectively^71,72^, and the rate of increase in metabolic rate below the LCT is substantially greater in water than in air (0.60 versus 0.17 W × kg^−1^ °C^−1^)^71^. Similarly, in another small diving seabird, the Cassin's auklet (*Ptychoramphus aleuticus*), the LCT in air and water were 16 and 21 °C, respectively, and resting metabolic rate was 25% higher in water than in air^73^. Although, comparable data for the little auk is unavailable, these species are in the same family (Alcidae) as the little auk, and share a similar ecology.

At the Ukaleqarteq study site, even in an exceptionally warm year (2021) with very low sea ice coverage (mean ± SE: 0.04 ± 0.02%), sea surface temperature within the foraging range (mean ± SE: 5.89 ± 0.38 °C)^15^ remained well below the LCTs in water reported for other Alcids (see above). Thus, even in warming oceans, little auks resting on the water may need to elevate their metabolic rate relative to when resting on ice, which, *in lieu* of compensatory changes, could elevate daily energy expenditure^74^, induce birds to return to the colony sooner, limiting time for energy acquisition, or force higher feeding rates. In addition, we recently demonstrated that high SST is associated with elevated daily energy expenditure in Ukaleqarteq little auks, a phenomenon associated with increased flight costs, as birds appeared to fly further to reach foraging grounds^75^. Longer flights may be motivated by the higher quality of lipid-rich copepods associated with colder ocean temperatures, but also by the opportunity to use sea ice as a resting substrate^15,75^. Loss of sea ice as a substrate for resting, foraging, and movement has demonstrated effects on energy balance in many sea ice-dependent species^76–78^. For instance, polar bears (*Ursus maritimus*) and narwhal (*Monodon monoceros*) show three–fourfold increases in locomotory costs in association with sea ice declines^78^.

T_b_ of little auks was also sensitive to environmental conditions. However, mean T_b_ was not related to T_a_. It is possible that a non-linear relationship could exist between T_a_ and T_b_, which we could not capture given our modeling approach. On the other hand, variation in T_b_ increased with T_a_ across behavioral states, which could indicate that these cold-adapted birds face increasing challenges maintaining stable T_b_ at higher temperatures, although, again, ambient temperature did not exceed the UCT of little auks in this study. T_b_ also increased with RH across behavioral states. As RH rises, capacity for evaporative heat dissipation decreases, resulting in increases in T_b_ or elevated thermoregulatory costs to maintain optimal T_b_^79,80^. In contrast, T_b_ tended to increase with wind speed when birds were in flight and decreased with wind speed when birds were diving. High winds have been associated with increased energetic costs of flight for many avian species with a flapping flight mode^81^, whereas T_b_ while foraging in cold waters could be further reduced by high winds via enhanced thermal conductance and heat loss. As for mean T_b_, wind speed and behavioral state interacted to predict between minute variation in T_b_. Specifically, between minute variation in T_b_ increased with wind speed when birds were flying and diving, while varying little with wind speed in the other behavioral states. A possible explanation for these results is that little auks have difficulty maintaining thermal stability when foraging in turbulent seas and flying in challenging conditions induced by higher winds. In the context of climate change, results suggest that alterations in RH and T_a_ may have implications for T_b_ regulation that are independent of behavioral state. On the other hand, changes in wind patterns may have especially high costs during active periods, with increases in storm events associated with climate change potentially elevating energy expenditure. As a caveat, weather conditions measured at the colony were taken as a proxy of conditions experienced by birds across behavioral states. Thus, given that little auks can forage up to ~ 100 km from the colony, results regarding effects of weather conditions on T_b_ should be interpreted with caution. Unfortunately, we did not have access to off-shore weather data on a fine temporal scale, and since birds were not GPS-tracked, we also had no way of knowing their precise locations during foraging trips.

We found no evidence for a relationship between Hg contamination and T_b_. However, our effective sample size for testing the relationship was low. In addition, little auks have lower Hg levels than many seabirds species that feed at higher trophic levels. Thus, our results regarding the relationship between Hg contamination and T_b_ are preliminary, and further research is needed in this area, perhaps utilizing a different species with higher contamination levels. To our knowledge, there is currently no study documenting a link between Hg concentrations and T_b_ in free-ranging animals. However, laboratory studies have demonstrated hypothermic responses to Hg exposure, for instance, in the mouse (*Mus musculus*)^82^. Hypothermic responses to contamination are hypothesized to reduce the toxicity of the chemical in the body^30^, but could create challenges for survival in dynamic thermal environments. We also observed weak, non-significant negative relationships between Hg levels and variation in T_b_, which is inconsistent with the hypothesis that contaminated bird have more difficulty maintaining stable T_b_.

Finally, there was a non-linear, bimodal relationship between time of day and T_b_. The highest values occurred in late morning and at night and the lowest in early morning and late afternoon. The pattern in T_b_ observed did not parallel daily cyclicity in T_a_ and RH, suggesting that it cannot be explained solely by diel variation in weather patterns. However, despite the fact that little auks in our population breed under 24-h of daylight, the pattern may reflect a combination of the timing of maximum solar radiation exposure, diel activity patterns, and/or underlying circadian rhythmicity in T_b_ independent of activity. A past study on little auks found a regular rhythm of population attendance at the population level, perhaps linked to variation in predation pressure, which provides some foundation for expecting that T_b_ could also display cyclic variation. However, this same study found little circadian rhythm in activity of individual little auks^83^. In contrast to mean T_b_, between minute variation in T_b_ did not correlate with time of day.

## Conclusions

T_b_ of little auks fluctuated according to behavioral state and environmental conditions, which likely aids animals in optimizing energy balance while performing essential behaviors in complex environments. Although this plasticity is predicted to facilitate energy balance in the face of climate change, the dynamic nature of T_b_ regulation also suggests that changing environmental conditions may significantly alter energy balance, or the behavioral and energetic strategies that must be adopted to achieve energetic homeostasis. Our data suggests that little auks use sea ice as a thermal refuge, resting on this substrate to allow T_b_ to rebound after submersion in cold water and ingestion of cold prey items. If sea ice decreases due to warming temperature, thermoregulatory costs are forecast to increase as birds are instead required to rest on the water surface, which may force restructuring of foraging strategies. No relationship was found between T_b_ of little auks and Hg concentrations, but our results are preliminary, and we call for more research on the effects of chemical contaminations on T_b_, especially in interaction with other environmental stressors.

### Supplementary Information

Supplementary Information.

## Data Availability

The datasets generated during this study are publicly available via the Zenodo community of European Commission Funded Research (OpenAIRE) online data repository (https://doi.org/10.5281/zenodo.7220883).

## References

[1] IPCC. Climate Change 2021: The Physical Science Basis. Contribution of Working Group I to the Sixth Assessment Report of the Intergovernmental Panel on Climate Change. Masson-Delmotte, V., Zhai, P., Pirani, A., Connors, S.L., Péan, C., *et al.* (eds.). Cambridge University Press (2021).

[2] Humphries MM (2004). Bioenergetic prediction of climate change impacts on Northern Mammals. Integr. Comp. Biol..

[3] Helmuth B (2009). From cells to coastlines: How can we use physiology to forecast the impacts of climate change?. J. Exp. Biol..

[4] Dillon ME, Wang G, Huey RB (2010). Global metabolic impacts of recent climate warming. Nature.

[5] Boyles JG, Seebacher F, Smit B, McKechnie AE (2011). Adaptive thermoregulation in endotherms may alter responses to climate change. Int. Comp. Biol..

[6] Cook TR, Martin R, Roberts J, Häkkinen H, Botha P (2020). Parenting in a warming world: Thermoregulatory responses to heat stress in an endangered seabird. Conserv. Physiol..

[7] Choy ES, O’Connor RS, Gilchrist HG, Hargreaves AL, Love (2021). Limited heat tolerance in a cold-adapted seabird: Implications of a warming Arctic. J. Exp. Biol..

[8] O’Connor RS, Le Pogam A, Young KG, Robitaille F, Choy (2021). Limited heat tolerance in an Arctic passerine: Thermoregulatory implications for cold-specialized birds in a rapidly warming world. Ecol. Evol..

[9] O’Connor RS, Le Pogam A, Young KG, Love OP, Cox CJ (2022). Warming in the land of the midnight sun: Breeding birds may suffer greater heat stress at high- versus low-Arctic sites. Proc. Roy. Soc. B.

[10] Wolf BO, Walsberg GE (1996). Thermal effects of radiation and wind on a small bird and implications for microsite selection. Ecology.

[11] Freeman MT, Czenze ZJ, Schoeman K, McKechnie AE (2022). Adaptive variation in the upper limits of avian body temperature. Proc. Natl. Acad. Sci..

[12] Kearney M, Shine R, Porter WP (2009). The potential for behavioral thermoregulation to buffer “cold-blooded” animals against climate warming. Proc. Natl. Acad. Sci..

[13] Rantanen M, Karpechko AY, Lipponen A, Nordling K, Hyvärinen O (2022). The Arctic has warmed nearly four times faster than the globe since 1979. Commun. Earth Environ..

[14] Amélineau F, Grémillet D, Harding AMA, Walkusz W, Choquet R (2019). Arctic climate change and pollution impact little auk foraging and fitness across a decade. Sci. Rep..

[15] Grunst AS, Grunst ML, Grémillet D, Kato A, Bustamante P (2023). Mercury contamination challenges the behavioral response of a keystone species to arctic climate change. Environ. Sci. Technol..

[16] AMAP, 2021. AMAP Mercury Assessment. Summary for Policy-makers. Arctic Monitoring and Assessment Programme (AMAP), Tromsø, Norway. 16 pp. (2021).

[17] Jenssen BM (2006). Endocrine-disrupting chemicals and climate change: A worst-case combination for arctic marine mammals and seabirds?. Environ. Health Persp..

[18] Hooper MJ, Ankley GT, Cristol DA, Maryoung LA, Noyes PD (2013). Interactions between chemical and climate stressors: A role for mechanistic toxicology in assessing climate change risks. Environ. Toxicol. Chem..

[19] Grunst AS, Grunst ML, Fort J (2023). Contaminant-by-environment interactive effects on animal behavior in the context of global change: Evidence from avian behavioral ecotoxicology. Sci. Tot. Environ..

[20] Morel FMM, Kraepiel AML, Amyot M (1998). The chemical cycle and bioaccumulation of mercury. Ann. Rev. Ecol. System..

[21] Jonsson S, Mastromonaco MN, Wang F, Bravo AG, Cairns WRL (2022). Arctic methylmercury cycling. Sci. Tot. Environ..

[22] Whitney, M. C., & Cristol, D. A. Impacts of Sublethal Mercury Exposure on Birds: A Detailed Review. In P. de Voogt (ed.), *Rev. Environ. Contam. Toxicol.,* **244**, 113–163. Springer. 10.1007/398_2017_4 (2017). 10.1007/398_2017_4 28710647

[23] Mckinney MA, Pedro S, Dietz R, Sonne C, Fisk AT (2015). A review of ecological impacts of global climate change on persistent organic pollutant and mercury pathways and exposures in arctic marine ecosystems. Curr. Zool..

[24] Cossa D (2013). Methylmercury manufacture. Nat. Geosci..

[25] Rice KM, Walker EM, Wu M, Gillette C, Blough ER (2014). Environmental mercury and its toxic effects. J. Prevent. Med. Pub. Health.

[26] Wada H, Cristol DA, McNabb FMA, Hopkins WA (2009). Suppressed adrenocortical responses and thyroid hormone levels in birds near a mercury-contaminated river. Environ. Sci. Technol..

[27] Calow P (1991). Physiological costs of combating chemical toxicants: Ecological implications. Comp. Biochem. Physiol. C.

[28] Gerson AR, Cristol DA, Seewagen CL (2019). Environmentally relevant methylmercury exposure reduces the metabolic scope of a model songbird. Environ. Pollut..

[29] Seewagen CL, Elowe CR, Gerson AR, Groom DJE, Ma Y (2022). Short-term mercury exposure disrupts muscular and hepatic lipid metabolism in a migrant songbird. Sci. Rep..

[30] Leon LR (2008). Thermoregulatory responses to environmental toxicants: The interaction of thermal stress and toxicant exposure. Toxicol. Appl. Pharmacol..

[31] Noyes PD, McElwee MK, Miller HD, Clark BW, Van Tiem LA (2009). The toxicology of climate change: Environmental contaminants in a warming world. Environ. Int..

[32] Ste-Marie E, Grémillet D, Fort J, Patterson A, Brisson-Curadeau É (2022). Accelerating animal energetics: High dive costs in a small seabird disrupt the dynamic body acceleration–energy expenditure relationship. J. Exp. Biol..

[33] Grémillet D, Kuntz G, Woakes AJ, Gilbert C, Robin J-P (2005). Year-round recordings of behavioural and physiological parameters reveal the survival strategy of a poorly insulated diving endotherm during the Arctic winter. J. Exp. Biol..

[34] Favilla AB, Costa DP (2020). Thermoregulatory strategies of diving air-breathing marine vertebrates: A review. Front. Ecol. Evol..

[35] Niizuma Y, Gabrielsen GW, Sato K, Watanuki Y, Naito Y (2007). Brünnich’s guillemots (Uria lomvia) maintain high temperature in the body core during dives. Comp. Biochem. Physiol. A.

[36] Egevang C, Boertmann D, Mosbech A, Tamstorf MP (2003). Estimating colony area and population size of little auks Alle alle at Northumberland Island using aerial images. Polar Biol..

[37] Wojczulanis-Jakubas K, Jakubas D, Stempniewicz L (2009). Sex-specific parental care by incubating little auks (*Alle alle*). Ornis Fenn..

[38] ^38^Montevecchi, W. A. & Stenhouse, I. J. (2020). Dovekie (*Alle alle*), version 1.0. In Birds of the World (Billerman, S. M., ed.) Cornell Lab of Ornithology. 10.2173/bow.doveki.01 (2020).

[39] Fort J, Robertson GJ, Grémillet D, Traisnel G, Bustamante P (2014). Spatial ecotoxicology: Migratory Arctic seabirds are exposed to mercury contamination while overwintering in the Northwest Atlantic. Environ. Sci. Technol..

[40] Carravieri A, Lorioux S, Angelier F, Chastel O, Albert C (2023). Carryover effects of winter mercury contamination on summer concentrations and reproductive performance in little auks. Environ. Pollut..

[41] Tattersall GJ, Roussel D, Voituron Y, Teulier L (2016). Novel energy-saving strategies to multiple stressors in birds: The ultradian regulation of body temperature. Proc. R. Soc. B.

[42] Gauchet L, Jaeger A, Grémillet D (2022). Using facial infrared thermography to infer avian body temperatures in the wild. Mar. Biol..

[43] Amélineau F, Grémillet D, Bonnet D, Le Bot T, Fort J (2016). Where to forage in the absence of sea ice? Bathymetry as a key factor for an Arctic seabird. PLOS ONE.

[44] Luque SP (2007). Diving behaviour analysis in R. R News.

[45] Sibly RM, Nott HMR, Fletcher DJ (1990). Splitting behaviour into bouts. Anim. Behav..

[46] Mori Y, Yoda K, Sato K (2001). Defining dive bouts using a sequential difference analysis. Behaviour.

[47] Chastel O, Fort J, Ackerman JT, Albert C, Angelier F (2022). Mercury contamination and potential health risks to Arctic seabirds and shorebirds. Sci. Tot. Environ..

[48] Bustamante P, Lahaye V, Durnez C, Churlaud C, Caurant F (2006). Total and organic Hg concentrations in cephalopods from the North Eastern Atlantic waters: Influence of geographical origin and feeding ecology. Sci. Tot. Environ..

[49] R Core Team. R: A language and environment for statistical computing. R Foundation for Statistical Computing, Vienna, Austria. https://www.R-project.org/. (2019).

[50] Wood SN (2011). Fast stable restricted maximum likelihood and marginal likelihood estimation of semiparametric generalized linear models: Estimation of Semiparametric Generalized Linear Models. J. R. Stat. Soc. B.

[51] Wood, S. N. Generalized Additive Models: An Introduction with R (2^nd^ edition). Chapman and Hall/CRC. (2017).

[52] Pinheiro, J., Bates, D., DebRoy, S., Sarkar, D., R Core Team. Nlme: Linear and Nonlinear Mixed Effects Models. R package version 3.1–143. https://CRAN.R-project.org/package=nlme. (2019).

[53] Schielzeth H (2010). Simple means to improve the interpretability of regression coefficients: *Interpretation of regression coefficients*. Meth. Ecol. Evo..

[54] Lenth, R. emmeans: Estimated Marginal Means, aka Least-Squares Means. R package version 1.4.3.01. https://CRAN.R-project.org/package=emmeans (2019).

[55] Lüdecke D (2018). ggeffects: Tidy data frames of marginal effects from regression models. J. Open Sour. Soft..

[56] Kwasniewski S, Gluchowska M, Jakubas D, Wojczulanis-Jakubas K, Walkusz W (2010). The impact of different hydrographic conditions and zooplankton communities on provisioning Little Auks along the West coast of Spitsbergen. Prog. Oceanog..

[57] Grémillet D, Welcker J, Karnovsky N, Walkusz W, Hall M (2012). Little auks buffer the impact of current Arctic climate change. Marine Ecology Progress Series.

[58] Kooyman GL, Ponganis PJ (1998). The physiological basis of diving depth: Birds and Mammals. Annu Rev Physiol.

[59] Williams CL, Ponganis PJ (2021). Diving physiology of marine mammals and birds: The development of biologging techniques. Philos. Trans. Roy. Soc. B.

[60] Wilson RP, Pütz K, Grémillet D, Culik BM, Kierspel M (1995). Reliability of stomach temperature changes in determining feeding characteristics of seabirds. J. Exp. Biol..

[61] Bevan RM, Butler PJ, Woakes AJ, Boyd IL (2002). The energetics of Gentoo Penguins, *Pygoscelis papua,* during the breeding season: *Penguin energy expenditure*. Funct. Ecol..

[62] Green JA, Butler PJ, Woakes AJ, Boyd IL (2003). Energetics of diving in macaroni penguins. J. Exp. Biol..

[63] Dawson, W. R., & O’Connor, T. P. Energetic Features of Avian Thermoregulatory Responses. In C. Carey (ed.), *Avian Energetics and Nutritional Ecology*. 85–124. Springer US. 10.1007/978-1-4613-0425-8_4 (1996).

[64] Gabrielsen GW, Taylor JRE, Konarzewski M, Mehlum F (1991). Field and laboratory metabolism and thermoregulation in dovekies (*Alle alle*). The Auk.

[65] Carere C, Welink D, Drent PJ, Koolhaas JM, Groothuis TGG (2001). Effect of social defeat in a territorial bird (*Parus major*) selected for different coping styles. Physiol. Behav..

[66] Dezecache G, Zuberbühler K, Davila-Ross M, Dahl CD (2017). Skin temperature changes in wild chimpanzees upon hearing vocalizations of conspecifics. R. Soc. Open.

[67] Knoch S, Whiteside MA, Madden JR, Rose PE, Fawcett TW (2022). Hot-headed peckers: Thermographic changes during aggression among juvenile pheasants ( *Phasianus colchicus* ). Philos. Trans. R. Soc. B.

[68] Enstipp MR, Grémillet D, Lorentsen S-H (2005). Energetic costs of diving and thermal status in European shags (*Phalacrocorax aristotelis* ). J. Exp. Biol..

[69] Oswald SA, Arnold JM (2012). Direct impacts of climatic warming on heat stress in endothermic species: Seabirds as bioindicators of changing thermoregulatory constraints. Int. Zool..

[70] Tremblay F, Whelan S, Choy ES, Hatch SA, Elliott KH (2022). Resting costs too: The relative importance of active and resting energy expenditure in a sub-arctic seabird. J. Exp. Biol..

[71] Croll DA, McLaren E (1993). Diving metabolism and thermoregulation in common and thick-billed murres. J. Comp. Physiol. B..

[72] Gabrielsen GW, Mehlum F, Karlsen HE (1988). Thermoregulation in four species of arctic seabirds. J. Comp. Physiol. B.

[73] Richman SE, Lovvorn JR (2011). Effects of air and water temperatures on resting metabolism of auklets and other diving birds. Physiol. Biochem. Zool..

[74] Lovvorn JR, Grebmeier JM, Cooper LW, Bump JK, Richman SE (2009). Modeling marine protected areas for threatened eiders in a climatically changing Bering Sea. Ecol. Appl..

[75] Grunst ML, Grunst AS, Grémillet D, Kato A, Bustamante P (2023). A keystone avian predator faces elevated energy expenditure in a warming Arctic. Ecology..

[76] Post E, Bhatt US, Bitz CM, Brodie JF, Fulton TL (2013). Ecological consequences of sea-ice decline. Science.

[77] Laidre KL, Atkinson S, Regehr EV, Stern HL, Born EW (2020). Interrelated ecological impacts of climate change on an apex predator. Ecol. Appl..

[78] Pagano AM, Williams TM (2021). Physiological consequences of Arctic sea ice loss on large marine carnivores: Unique responses by polar bears and narwhals. J. Exp. Biol..

[79] Dawson WR (1982). Evaporative losses of water by birds. Comp. Biochem. Physiol. A.

[80] Gerson AR, Smith EK, Smit B, McKechnie AE, Wolf BO (2014). The impact of humidity on evaporative cooling in small desert birds exposed to high air temperatures. Physiol. Biochem. Zool..

[81] Elliott KH, Chivers LS, Bessey L, Gaston AJ, Hatch SA (2014). Windscapes shape seabird instantaneous energy costs but adult behavior buffers impact on offspring. Mov. Ecol..

[82] Gordon CJ, Fogelson L, Highfill JW (1990). Hypothermia and hypometabolism: Sensitive indices of whole-body toxicity following exposure to metallic salts in the mouse. J. Toxicol. Environ. Health.

[83] Wojczulanis-Jakubas K, Wąż P, Jakubas D (2020). Little auks under the midnight sun: Diel activity rhythm of a small diving seabird during the Arctic summer. Pol. Res..

