# Author Correction: Keystone seabird may face thermoregulatory challenges in a warming Arctic

**DOI:** 10.1038/s41598-023-47972-2

**Published:** 2023-11-22

**Authors:** Melissa L. Grunst, Andrea S. Grunst, David Grémillet, Akiko Kato, Sophie Gentès, Jérôme Fort

**Affiliations:** 1 Littoral, Environnement et Sociétés (LIENSs), UMR 7266 CNRS-La Rochelle Université, 2 Rue Olympe de Gouges, 17000 La Rochelle, France; 2 CEFE, Univ Montpellier, CNRS, EPHE, IRD, Montpellier, France; 3 Percy FitzPatrick Institute of African Ornithology, University of Cape Town, Rondebosch, South Africa; 4 Centre d’Etudes Biologiques de Chizé, CEBC, UMR 7372 CNRS-La Rochelle Université, La Rochelle, France

Correction to: *Scientific Reports* 10.1038/s41598-023-43650-5, published online 04 October 2023

The original version of this Article contained an error in Affiliation 2.

Affiliation 2

“CEFE, UMR 5175, CNRS - Université de Montpellier - Université Paul-Valéry Montpellier - EPHE, Montpellier, France”

now reads

Affiliation 2

“CEFE, Univ Montpellier, CNRS, EPHE, IRD, Montpellier, France”

The original Article has been corrected.

