# Supplementary material for: Keystone seabird may face thermoregulatory challenges in a warming Arctic
Source: Sci Rep. 2023 Oct 4;13:16733. doi: 10.1038/s41598-023-43650-5 (PMC10550970; doi:10.1038/s41598-023-43650-5)
Supplement: Supplementary file 1 — Supplementary Information. [file 41598_2023_43650_MOESM1_ESM.docx]

**Supplementary Material**

**Keystone seabird may face thermoregulatory challenges in a warming Arctic**

**Melissa L. Grunst^a*^, Andrea S. Grunst^a^, David Grémillet^b,c^, Akiko Kato^d^, Sophie Gentès^a^, Jérôme Fort^a^**

^a^Littoral, Environnement et Sociétés (LIENSs), UMR 7266 CNRS-La Rochelle Université, 2 Rue Olympe de Gouges, FR-17000 La Rochelle, France

^b^CEFE, UMR 5175, CNRS – Université de Montpellier – Université Paul-Valéry Montpellier – EPHE; Montpellier, France

^c^Percy FitzPatrick Institute of African Ornithology, University of Cape Town, Rondebosch, South Africa

^d^Centre d’Etudes Biologiques de Chizé, CEBC, UMR 7372 CNRS-La Rochelle Université, France

*Correspondance: mgrun002@ucr.edu

**Caption**: Global environmental change is transforming ecosystems at unprecedented rates, challenging organismal homeostasis. In this manuscript, we explore how variation in the body temperature (T_b_) of a keystone Arctic seabird, the little auk (*Alle alle*), is affected by climate change-sensitive environmental variables, declining sea ice habitat, and mercury (Hg) contamination. We evaluated the environment-dependency of T_b_ across five behavioral states (flying, foraging (diving), at the colony, on sea ice, resting on the water), as assessed through 3D accelerometry. In this supplement, we present results of statistical analyses that are too extensive for inclusion in the main text (Table S1-S7). In addition, we include graphs (Fig. S1-S7) showing how mean T_b_ changes through time, and color-coded with respect to behavioral state for 7 of our 8 focal birds (the graph for the 8^th^ individual is included as a representative in the main manuscript). Finally, Fig. S8 and S9 show the relationship between time of day and ambient temperature and relative humidity, respectively.

**Table S1**. Full GAMM for body temperature (T_b_; °C) in little auks as a function of behavioral state, weather conditions, mercury contamination and time.

| ***Parametric coefficients*** | |  |  |  |  |
| --- | --- | --- | --- | --- | --- |
| **Variable** | ***β* ± SE** | ***t*-value** | ***P* (> \|t\|)** | ***F*** | ***P* (> F)** |
| Intercept | 40.6 ± 0.464 | 87.5 | < 0.001 |  |  |
| Flying | 0.919 ± 0.495 | 1.857 | 0.063 | 1.772 | 0.131 |
| Colony | 0.821 ± 0.462 | 1.779 | 0.075 |  |  |
| Ice | 0.323 ± 0.468 | 0.691 | 0.489 |  |  |
| Water | 0.692 ± 0.494 | 1.402 | 0.161 |  |  |
| Wind speed (m/s) | -0.018 ± 0.007 | -2.708 | 0.007 | 12.445 | < 0.001 |
| Humidity (%) | 0.037 ± 0.026 | 1.416 | 0.157 | 0.162 | 0.687 |
| Hg (µg g^-1^) | 0.015 ± 0.064 | 0.235 | 0.815 | 4.840 | 0.027 |
| Temperature (°C) | -0.043 ± 0.024 | -1.808 | 0.071 | 3.181 | 0.074 |
| Flying × wind | 0.031 ± 0.010 | 3.126 | 0.002 | 5.338 | < 0.001 |
| Colony × wind | 0.021 ± 0.010 | 2.008 | 0.045 |  |  |
| Ice × wind | 0.024 ± 0.010 | 2.374 | 0.018 |  |  |
| Water × wind | 0.019 ± 0.012 | 1.612 | 0.107 |  |  |
| Flying × Humidity | 0.054 ± 0.036 | 1.506 | 0.132 | 0.193 | 0.942 |
| Colony × Humidity | 0.023 ± 0.038 | 0.601 | 0.548 |  |  |
| Ice × Humidity | 0.012 ± 0.038 | 0.304 | 0.761 |  |  |
| Water × Humidity | 0.029 ± 0.043 | 0.684 | 0.494 |  |  |
| Flying × Hg | 0.041 ± 0.034 | 1.221 | 0.222 | 1.747 | 0.136 |
| Colony × Hg | 0.066 ± 0.044 | 1.501 | 0.133 |  |  |
| Ice × Hg | 0.014 ± 0.044 | 0.313 | 0.754 |  |  |
| Water × Hg | -0.008 ± 0.036 | -0.213 | 0.831 |  |  |
| Flying × Temperature | 0.061 ± 0.033 | 1.829 | 0.067 | 0.520 | 0.721 |
| Colony × Temperature | 0.024 ± 0.035 | 0.69 | 0.490 |  |  |
| Ice × Temperature | 0.053 ± 0.034 | 1.549 | 0.121 |  |  |
| Water × Temperature | 0.051 ± 0.038 | 1.318 | 0.187 |  |  |
| ***Approximate significance, Smoothed terms*** | | |  |  |  |
|  | ***edf*** | ***Ref. df*** | ***F*** | ***P* *(< F)*** |  |
| s(Time behavior):Diving | 9.43 | 9.43 | 67.5 | < 0.001 |  |
| s(Time behavior):Flying | 5.95 | 5.95 | 10.0 | < 0.001 |  |
| s(Time behavior):Colony | 5.825 | 5.825 | 21.7 | < 0.001 |  |
| s(Time behavior):Ice | 5.086 | 5.086 | 21.3 | < 0.001 |  |
| s(Time behavior):Water | 2.097 | 2.097 | 0.707 | 0.461 |  |
| s(Time of day) | 6.372 | 18 | 3.809 | < 0.001 |  |
| **R^2^-adjusted** | 0.225 | **N** | 16405,8 |  |  |

**Table S2**. Estimated marginal means (EMMs) from the GAMM (df =16359) predicting little auk body temperature (T_b_; °C) within different behavior states, with pairwise contrasts.

| **Behavior** | **EMM ± SE (95% CI)** | | |
| --- | --- | --- | --- |
| Diving (D) | 40.65 ± 0.092 (40.47, 40.83) | | |
| Flying (F) | 41.55 ± 0.091 (41.37, 41.73) | | |
| Colony (C) | 41.37 ± 0.089 (41.20, 41.55) | | |
| Ice (I) | 41.39 ± 0.088 (41.22, 41.56) | | |
| Water (W) | 41.37 ± 0.104 (41.16, 41.58) | | |
| **Pairwise contrast** | ***β* ± SE** | ***t*** | ***P (>\|t\|*)** |
| D-F | -0.13 ± 0.04 | -3.5 | 0.005 |
| D-C | -0.47 ± 0.05 | -9.1 | <0.001 |
| D-I | 0.21 ± 0.05 | 4.3 | <0.001 |
| D-W | 0.03 ± 0.04 | 0.70 | 0.956 |
| F-C | -0.34 ± 0.05 | -6.9 | <0.001 |
| F-I | 0.33 ± 0.05 | 6.8 | <0.001 |
| F-W | 0.16 ± 0.04 | 3.8 | 0.002 |
| C-I | 0.68 ± 0.06 | 11.2 | <0.001 |
| C-W | 0.50 ± 0.06 | 8.9 | <0.001 |
| I-W | -0.18 ± 0.05 | -3.4 | 0.006 |

**Table S3**. Estimated marginal means (EMMs) from the GAMM (df =1115) predicting ΔT_b_ (°C) of little auks in the five behavioral states, with pairwise contrasts between behavioral states.

| **Behavior** | **EMM ± SE (95 % CI)** | | |
| --- | --- | --- | --- |
| Diving (D) | -0.312 ± 0.026 (-0.374, -0.251) | | |
| Flying (F) | 0.146 ± 0.029 (0.077, 0.215) | | |
| Colony (C) | -0.074 ± 0.044 (-0.179, 0.031) | | |
| Ice (I) | 0.335 ± 0.045 (0.228, 0.443) | | |
| Water (W) | 0.0002 ± 0.038 (-0.089, 0.089) | | |
| **Pairwise contrast** | ***β* ± SE** | ***t*** | ***P (>\|t\|*)** |
| D-F | -0.459 ± 0.045 | -10.29 | <0.001 |
| D-C | -0.238 ± 0.050 | -4.747 | <0.001 |
| D-I | -0.648 ± 0.055 | -11.70 | <0.001 |
| D-W | -0.313 ± 0.049 | -6.383 | <0.001 |
| F-C | 0.221 ± 0.058 | 3.737 | 0.002 |
| F-I | -0.189 ± 0.055 | -3.463 | 0.005 |
| F-W | 0.146 ± 0.049 | 2.991 | 0.024 |
| C-I | -0.409 ± 0.063 | -6.546 | <0.001 |
| C-W | -0.074 ± 0.058 | -1.273 | 0.708 |
| I-W | 0.335 ± 0.064 | 5.212 | <0.001 |

**Table S4.** Estimated marginal trends (EMTs) from the GAMM (df = 16357) for the effects of wind speed on body temperature (T_b_; °C) in little auks within different behavioral states, with pairwise contrasts.

| **Behavior** | **EMT ± SE (95% CI)** | | |
| --- | --- | --- | --- |
| Diving (D) | -0.016 ± 0.007 (-0.029, -0.003) | | |
| Flying (F) | 0.012 ± 0.007 (-0.002, 0.025) | | |
| Colony (C) | 0.004 ± 0.008 (-0.011, 0.019) | | |
| Ice (I) | 0.005 ± 0.007 (-0.009, 0.019) | | |
| Water (W) | 0.0003 ± 0.009 (-0.018, 0.019) | | |
| **Pairwise contrast** | ***β* ± SE** | ***t-ratio*** | ***P (>\|t\|*)** |
| D-F | -0.028 ± 0.010 | -2.894 | 0.031 |
| D-C | -0.021 ± 0.010 | -2.067 | 0.235 |
| D-I | -0.022 ± 0.010 | -2.232 | 0.168 |
| D-W | -0.017 ± 0.011 | -1.457 | 0.590 |
| F-C | 0.007 ± 0.011 | 0.701 | 0.956 |
| F-I | 0.006 ± 0.010 | 0.634 | 0.969 |
| F-W | 0.012 ± 0.012 | 0.975 | 0.866 |
| C-I | -0.001 ± 0.010 | -0.087 | 1.000 |
| C-W | 0.004 ± 0.012 | 0.344 | 0.997 |
| I-W | 0.005 ± 0.012 | 0.429 | 0.993 |

**Table S5**. Full GAMM predicting between minute variation in body temperature (|T_b1_-T_b1+1_|) (°C) in little auks as a function of behavioral state, weather conditions, mercury contamination, and time.

| ***Parametric coefficients*** | |  |  |  |  |
| --- | --- | --- | --- | --- | --- |
| **Variable** | ***β* ± SE** | ***t*-value** | ***P* (> \|t\|)** | ***F*** | ***P* (> F)** |
| Intercept | 0.155 ± 0.037 | 4.251 | < 0.001 |  |  |
| Flying | -0.039 ± 0.053 | -0.726 | 0.467 | 1.772 | 0.131 |
| Colony | -0.076 ± 0.037 | -2.076 | 0.038 |  |  |
| Ice | -0.091 ± 0.038 | -2.399 | 0.016 |  |  |
| Water | -0.070 ± 0.082 | -0.855 | 0.393 |  |  |
| Wind speed (m/s) | 0.009 ± 0.002 | 3.528 | < 0.001 | 12.445 | < 0.001 |
| Humidity (%) | -0.002 ± 0.006 | -0.403 | 0.687 | 0.162 | 0.687 |
| Hg (µg g^-1^) | -0.010 ± 0.005 | -2.2 | 0.028 | 4.840 | 0.027 |
| Temperature (°C) | 0.011 ± 0.006 | 1.784 | 0.074 | 3.181 | 0.074 |
| Flying × wind | 0.001 ± 0.004 | 0.15 | 0.881 | 5.338 | < 0.001 |
| Colony × wind | -0.010 ± 0.004 | -2.545 | 0.011 |  |  |
| Ice × wind | -0.010 ± 0.004 | -2.496 | 0.012 |  |  |
| Water × wind | -0.016 ± 0.005 | -3.485 | < 0.001 |  |  |
| Flying × Humidity | 0.003 ± 0.008 | 0.382 | 0.702 | 0.193 | 0.942 |
| Colony × Humidity | 0.006 ± 0.010 | 0.728 | 0.467 |  |  |
| Ice × Humidity | -0.002 ± 0.011 | -0.163 | 0.871 |  |  |
| Water × Humidity | 0.002 ± 0.012 | 0.189 | 0.850 |  |  |
| Flying × Hg | -0.0002 ± 0.005 | -0.056 | 0.956 | 1.747 | 0.136 |
| Colony × Hg | -0.001 ± 0.006 | -0.147 | 0.883 |  |  |
| Ice × Hg | 0.009 ± 0.005 | 1.6 | 0.110 |  |  |
| Water × Hg | 0.010 ± 0.005 | 1.899 | 0.058 |  |  |
| Flying × Temperature | -0.003 ± 0.008 | -0.397 | 0.691 | 0.520 | 0.721 |
| Colony × Temperature | -0.004 ± 0.008 | -0.51 | 0.610 |  |  |
| Ice × Temperature | -0.015 ± 0.010 | -1.422 | 0.155 |  |  |
| Water × Temperature | -0.003 ± 0.011 | -0.234 | 0.815 |  |  |
| ***Approximate significance, Smoothed terms*** | | |  |  |  |
|  | ***edf*** | ***Ref. df*** | ***F*** | ***P* *(< F)*** |  |
| s(Time behavior):Diving | 6.326 | 6.326 | 66.15 | < 0.001 |  |
| s(Time behavior):Flying | 6.674 | 6.674 | 50.73 | < 0.001 |  |
| s(Time behavior):Colony | 11.644 | 11.644 | 15.51 | < 0.001 |  |
| s(Time behavior):Ice | 5.081 | 5.081 | 17.29 | < 0.001 |  |
| s(Time behavior):Water | 3.354 | 3.354 | 40.63 | < 0.001 |  |
| s(Time of day) | 1.198 | 8.000 | 0.297 | 0.113 |  |
| **R^2^-adjusted** | 0.234 | **N** | 15136,8 |  |  |

**Table S6**. Estimated marginal means (EMMs) from the GAMM (df =15092) for between minute variation in little auk body temperature (|T_b1_-T_b1+1_|) (°C) within different behavior states, with pairwise contrasts.

| **Behavior** | **EMM ± SE (95% CI)** | | |
| --- | --- | --- | --- |
| Diving (D) | 0.097 ± 0.008 (0.080, 0.113) | | |
| Flying (F) | 0.060 ± 0.008 (0.044, 0.077) | | |
| Colony (C) | 0.041 ± 0.008 (0.024, 0.057) | | |
| Ice (I) | 0.040 ± 0.007 (0.025, 0.054) | | |
| Water (W) | 0.055 ± 0.011 (0.033, 0.078) | | |
| **Pairwise contrast** | ***β* ± SE** | ***t-ratio*** | ***P (>\|t\|*)** |
| D-F | 0.036 ± 0.010 | 3.512 | 0.004 |
| D-C | 0.056 ± 0.010 | 5.367 | < 0.0001 |
| D-I | 0.057 ± 0.009 | 5.947 | < 0.0001 |
| D-W | 0.041 ± 0.013 | 3.171 | 0.013 |
| F-C | 0.020 ± 0.010 | 1.906 | 0.314 |
| F-I | 0.021 ± 0.009 | 2.196 | 0.181 |
| F-W | 0.005 ± 0.013 | 0.383 | 0.995 |
| C-I | 0.001 ± 0.010 | 0.111 | 1.000 |
| C-W | -0.015 ± 0.013 | -1.131 | 0.790 |
| I-W | -0.015 ± 0.012 | -1.278 | 0.705 |

**Table S7.** Estimated marginal trends (EMTs) from the GAMM (df = 15092) for the effects of wind speed on between minute variation in body temperature in little auks (|T_b1_-T_b1+1_|) (°C) within different behavioral states, with pairwise contrasts.

| **Behavior** | **EMT ± SE (95% CI)** | | |
| --- | --- | --- | --- |
| Diving (D) | 0.008 ± 0.002 (0.004, 0.013) | | |
| Flying (F) | 0.009 ± 0.003 (0.003, 0.014) | | |
| Colony (C) | -0.001 ± 0.003 (-0.007, 0.004) | | |
| Ice (I) | -0.001 ± 0.003 (-0.006, 0.005) | | |
| Water (W) | -0.007 ± 0.004 (-0.015, 0.0003) | | |
| **Pairwise contrast** | ***β* ± SE** | ***t-ratio*** | ***P (>\|t\|*)** |
| D-F | -0.0002 ± 0.004 | -0.077 | 1.000 |
| D-C | 0.010 ± 0.004 | 2.598 | 0.071 |
| D-I | 0.009 ± 0.004 | 2.317 | 0.139 |
| D-W | 0.016 ± 0.004 | 3.415 | 0.009 |
| F-C | 0.010 ± 0.004 | 2.586 | 0.073 |
| F-I | 0.009 ± 0.004 | 2.313 | 0.141 |
| F-W | 0.016 ± 0.005 | 3.398 | 0.006 |
| C-I | -0.001 ± 0.004 | -0.161 | 0.999 |
| C-W | 0.006 ± 0.005 | 1.278 | 0.705 |
| I-W | 0.007 ± 0.005 | 1.379 | 0.641 |


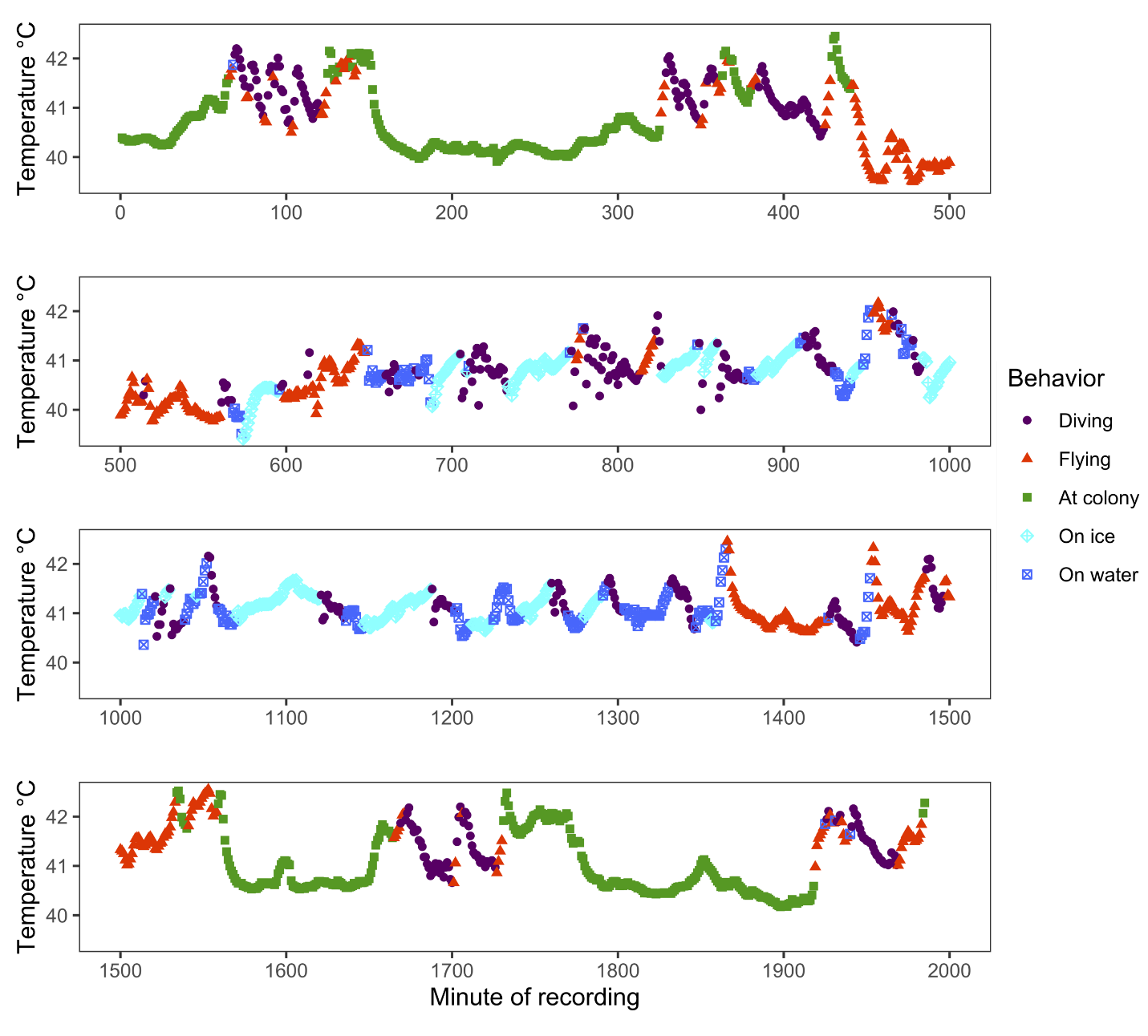


**Figure S1**. Variation in body temperature (T_b_**)** through time and color coded with respect to behavioral state for one individual little auk (LIAK20EG09) from the Ukaleqarteq, East Greenland, population. In this individual, decreases in T_b_ while diving, followed by rebounds in T_b_ on the sea ice are clearly notable, and T_b_ also appears to decrease with time at the colony. Although the overall pattern is for T_b_ to tend to increase with time in flight, in this individual, there are also some instances wherein T_b_ appears to decrease while flying. Across individuals, such instances appear most common when birds are departing the colony (e.g. around 450 min), and could reflect exposure to colder air temperatures and wind over the sea relative to over land.


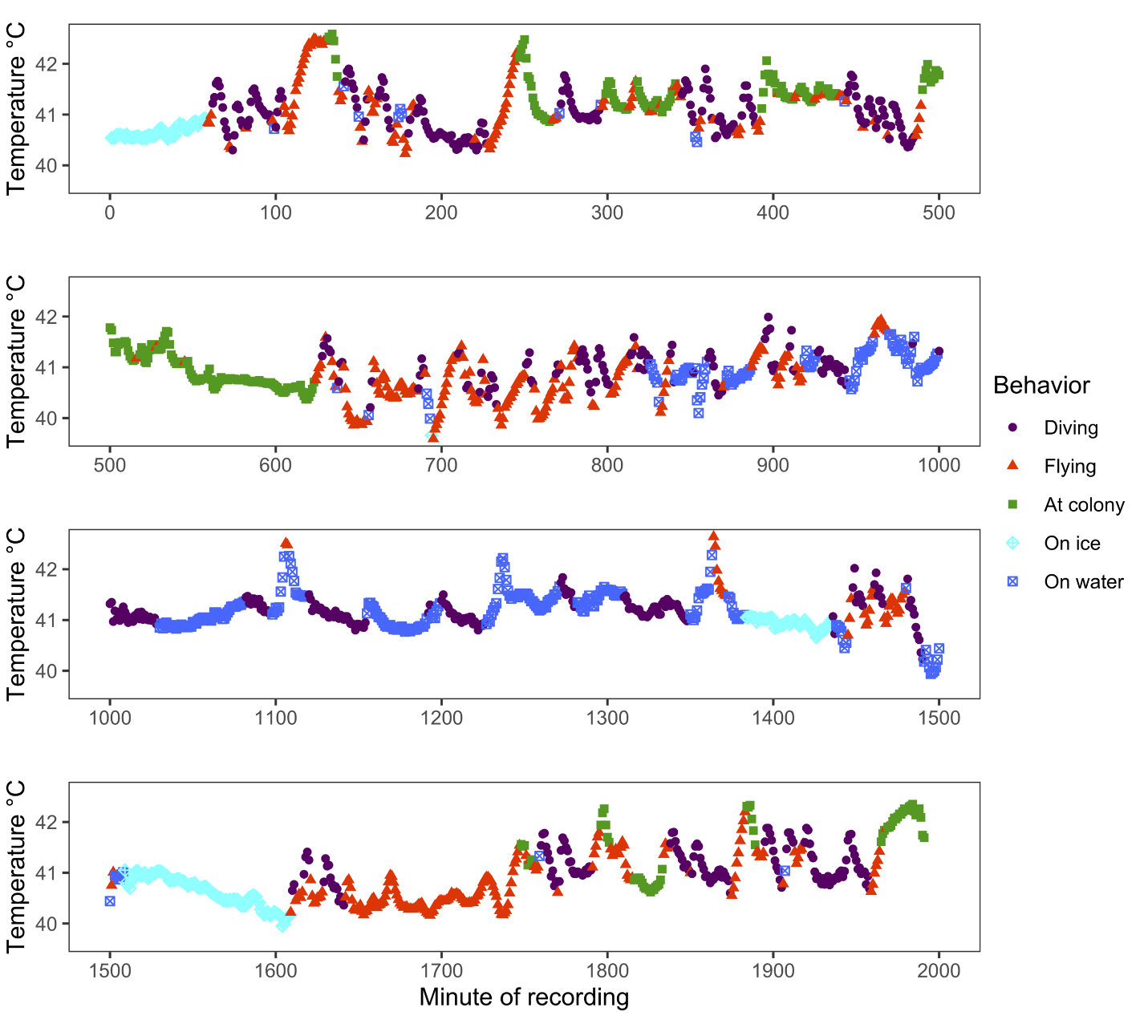


**Figure S2**. Variation in body temperature (T_b_**)** through time and color coded with respect to behavioral state for one individual little auk (LIAK20EG12) from the Ukaleqarteq, East Greenland, population. In this individual, the time spent on sea ice does not immediately follow diving bouts, so the rebound in T_b_ on sea ice, which observed in other individuals, is not apparent. Notably, rather than spending time on sea ice in between diving, the individual spent more time on water, and it can be observed that T_b_ does recover as well while the bird is resting on the water, relative to on sea ice. T_b_ can be observed to generally increase during flight, and decrease while at the colony.


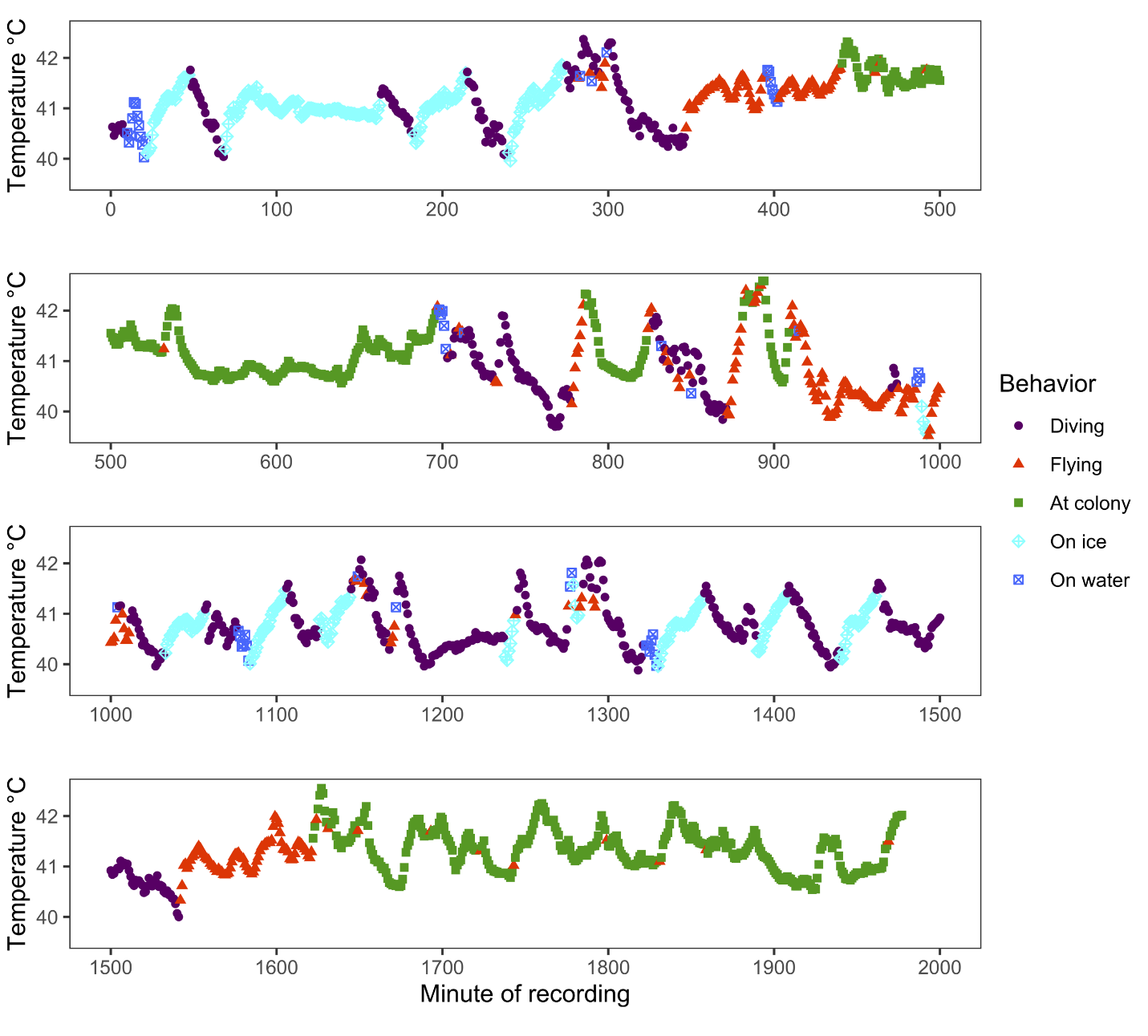


**Figure S3**. Variation in body temperature (T_b_**)** through time and color coded with respect to behavioral state for one individual little auk (LIAK20EG14) from the Ukaleqarteq, East Greenland, population. Note the rebound in T_b_ on sea ice following declines during diving. T_b_ can be observed to increase while flying in several cases, but again T_b_ appears to decrease in flight after leaving the colony, which may reflect departure from a thermal refuge (see also Fig. S1). In this individual, we observe some variability in T_b_ at the colony, associated with interspersion of short flying episodes. Little auks often take flight at the colony when threatened by predators (especially glaucous gulls (*Larus hyperboreus*)), and associated activation of stress responses may result in elevation of, and variation in, T_b._


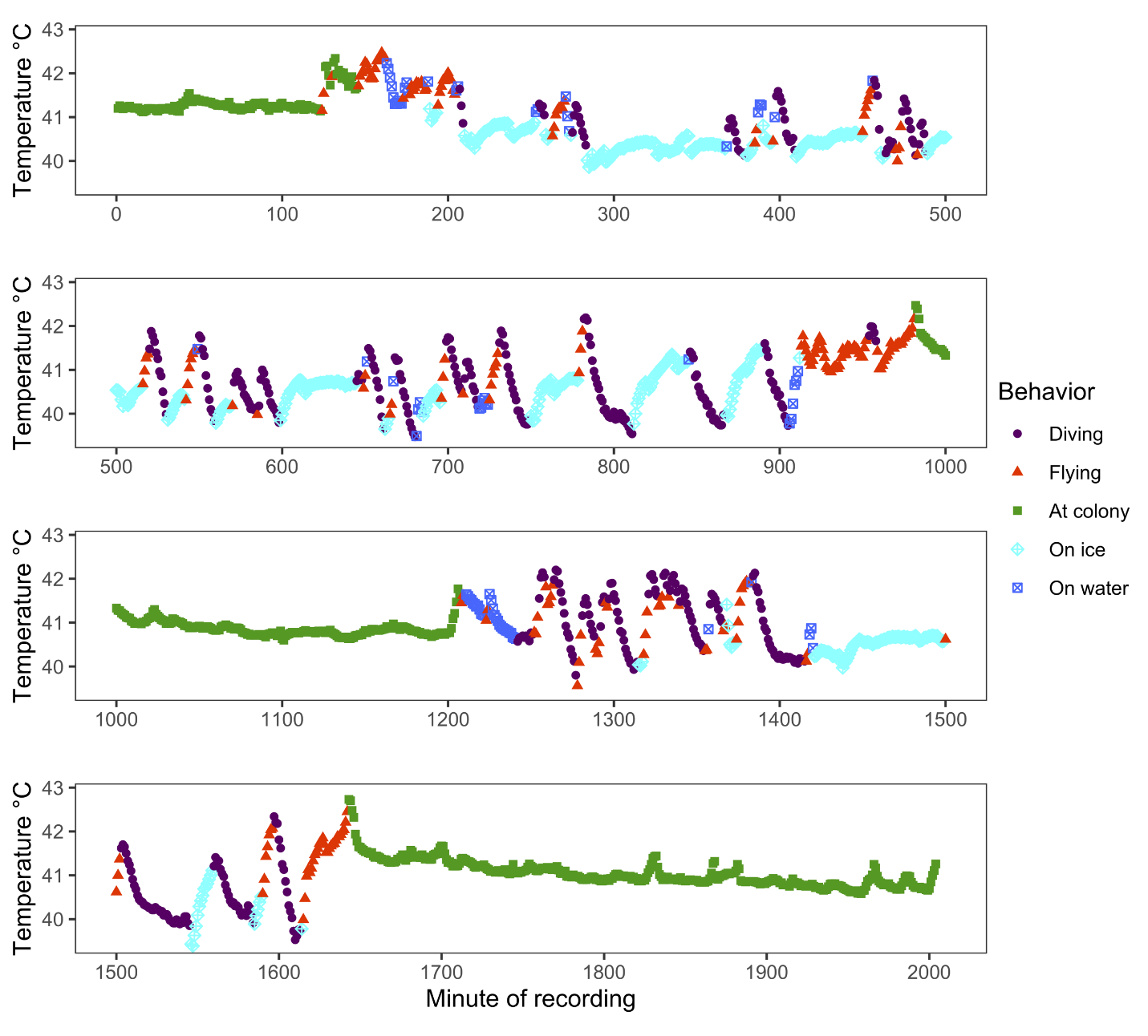


**Figure S4**. Variation in body temperature (T_b_**)** through time and color coded with respect to behavioral state for one individual little auk (LIAK20EG17) from the Ukaleqarteq, East Greenland, population. Note rebounds in T_b_ when on the sea ice following declines while diving in cold polar waters. T_b_  can also be observed to increase with time during flight and decline with time at the colony. Overall stability of T_b_ at the colony is also apparent.


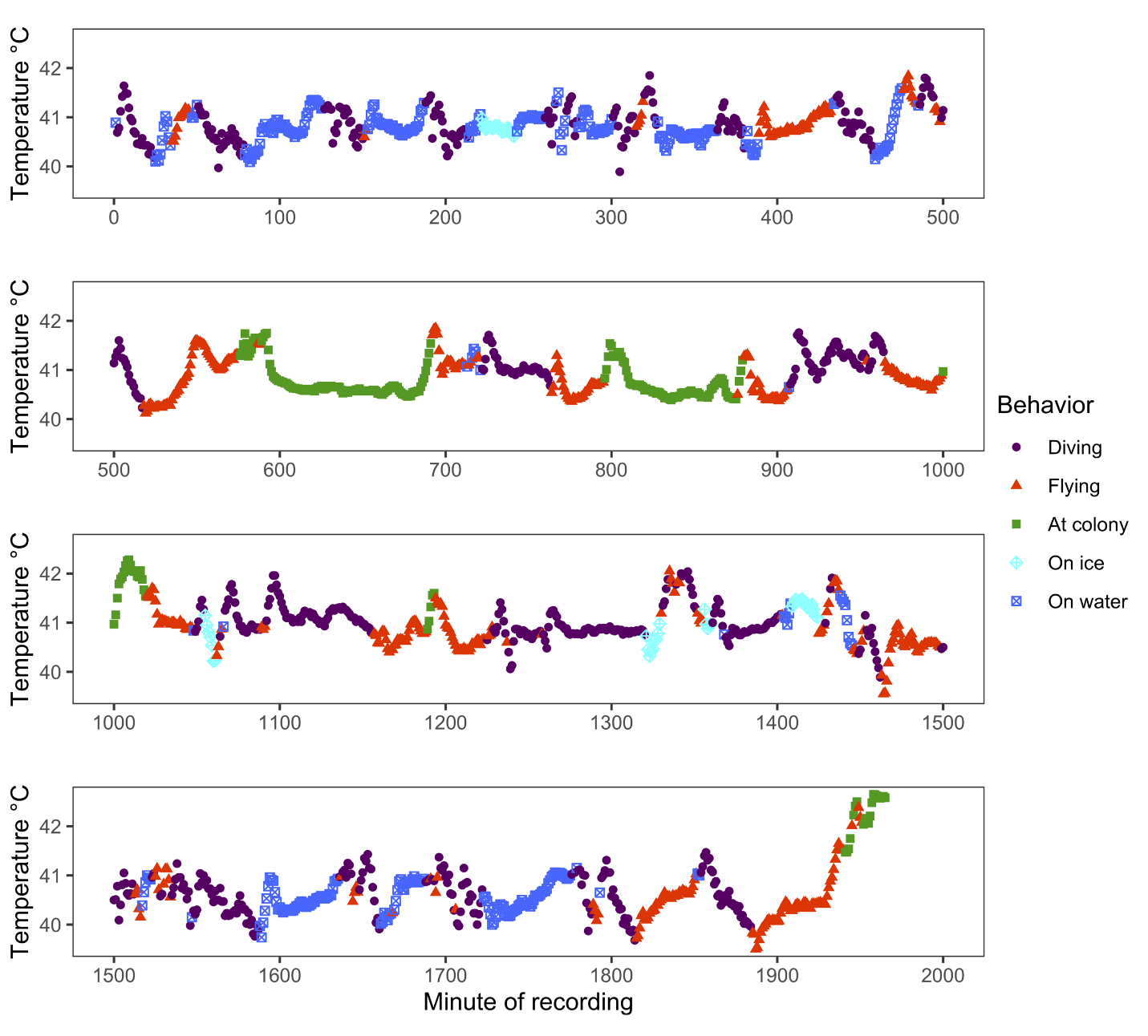


**Figure S5**. Variation in body temperature (T_b_**)** through time and color coded with respect to behavioral state for one individual little auk (LIAK20EG24) from the Ukaleqarteq, East Greenland, population. This bird spent little time on ice, and the rebound in T_b_ on ice following diving bouts is not clear. T_b_ can be observed to increase during flight in several instances, and to decline at the colony. While resting on the water surface, the pattern of change in T_b_ with time appears variable.


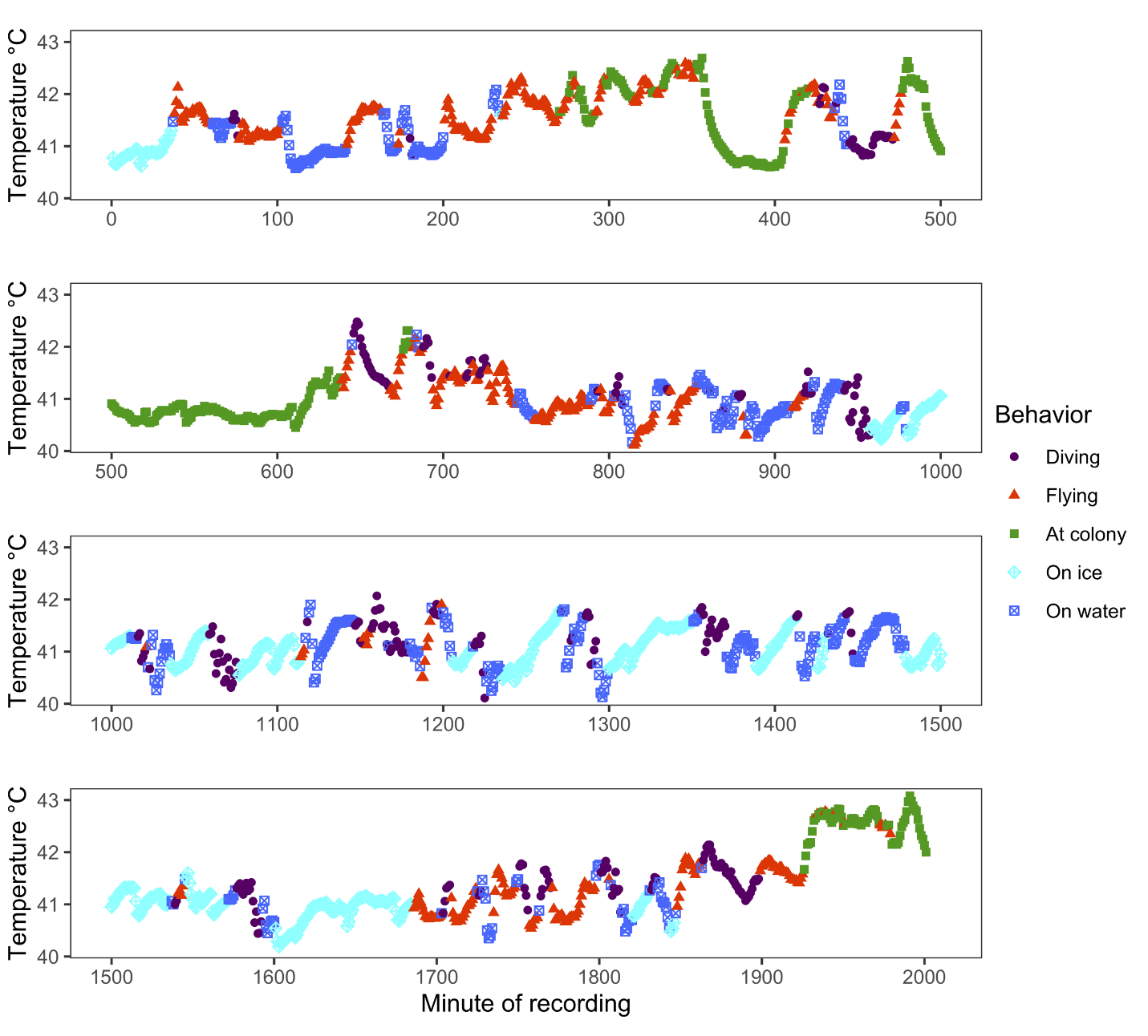


**Figure S6**. Variation in body temperature (T_b_**)** through time and color coded with respect to behavioral state for one individual little auk (LIAK20EG25) from the Ukaleqarteq, East Greenland, population. In this bird, the rebound in T_b_ on ice following foraging bouts (diving) is apparent. T_b_ can also be observed to increase during flight in serval instances. Interspersion of short flight episodes with time at the colony can be observed to increase variability of T_b_ at the colony (e.g. ~minute 275-350).


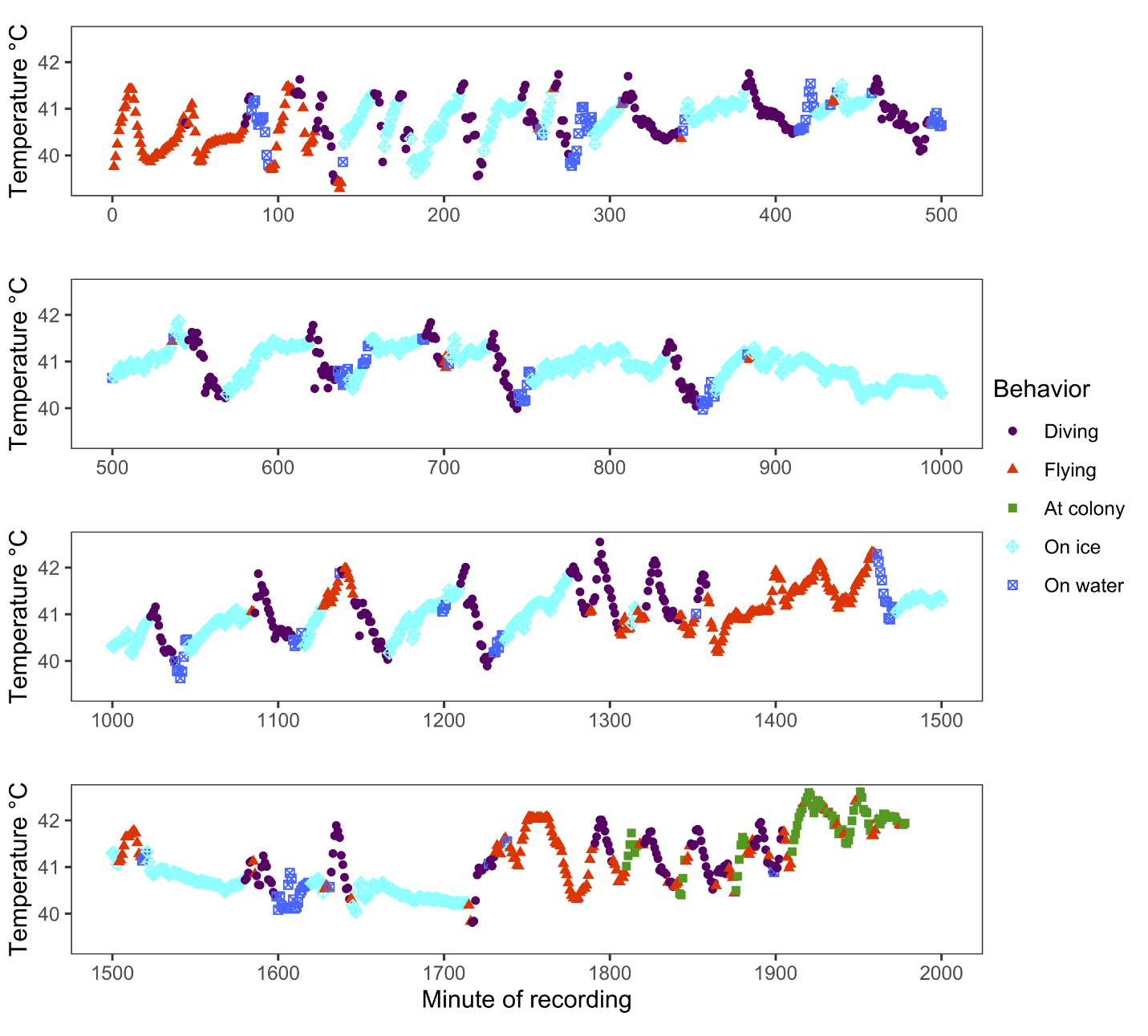


**Figure S7**. Variation in body temperature (T_b_**)** through time and color coded with respect to behavioral state for one individual little auk (LIAK20EG28) from the Ukaleqarteq, East Greenland, population. The rebound of T_b_ on ice following foraging bouts (diving) is apparent. In this individual the pattern of T_b_ variation through time in flight shows some increases and decreases. The decreases are somewhat difficult to explain in these cases, but could reflect changes in exposure to wind.


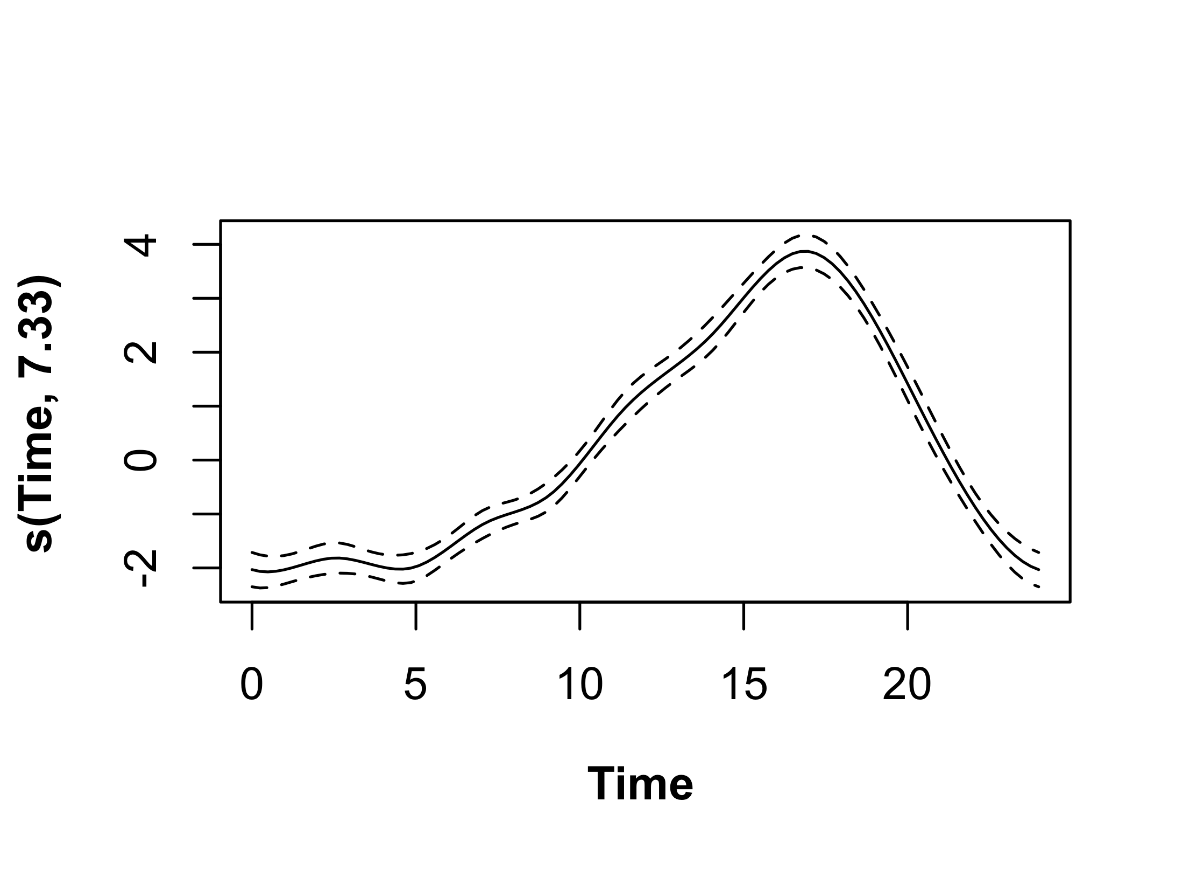


**Figure S8**. Residuals from the GAMM predicting ambient temperature from time of day, with a smooth curve fitted. Dashed lines show 2-SE limits. The y-axis shows the partial effect of time of day on ambient temperature.


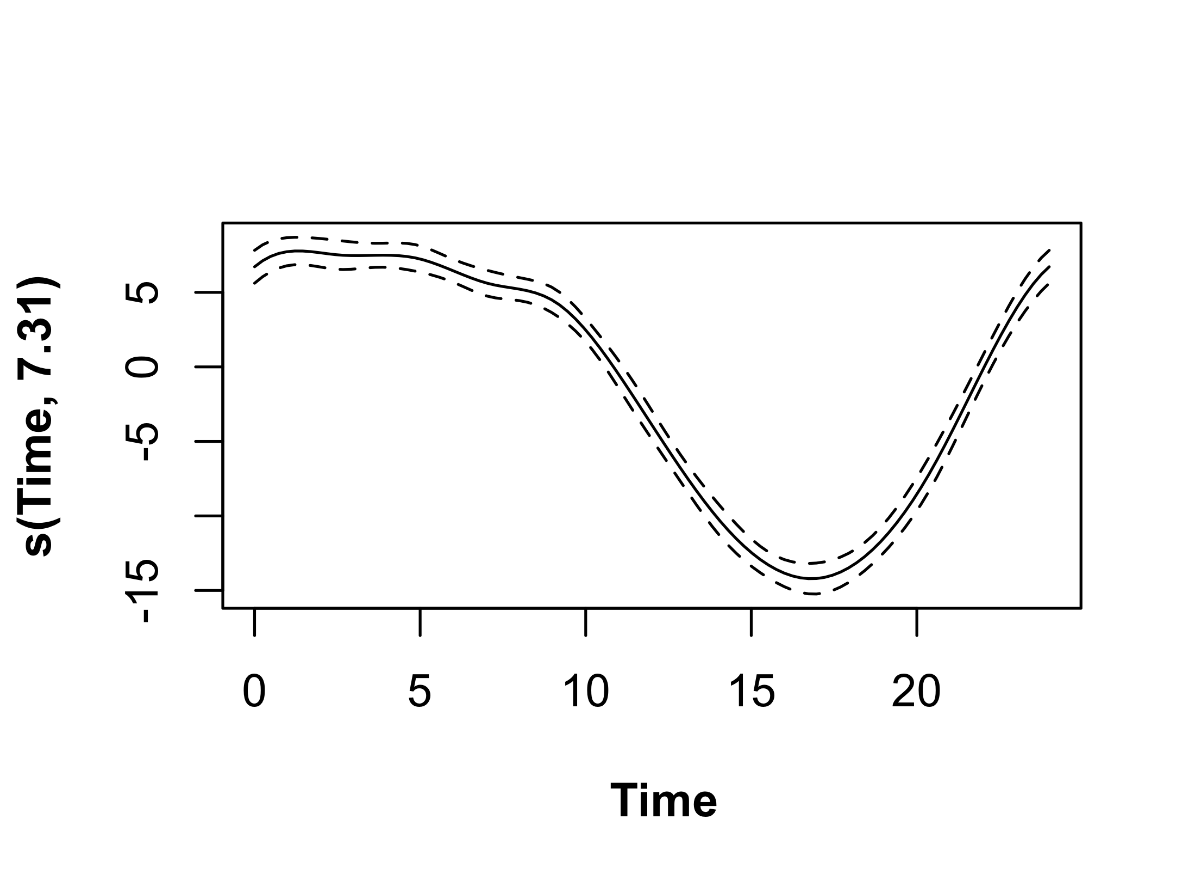


**Figure S9**. Residuals from the GAMM predicting relative humidity from time of day, with a smooth curve fitted. Dashed lines show 2-SE limits. The y-axis shows the partial effect of time of day on relative humidity.
